# GABAergic synapses: their plasticity and role in sensory cortex

**DOI:** 10.3389/fncel.2014.00091

**Published:** 2014-03-26

**Authors:** Trevor C. Griffen, Arianna Maffei

**Affiliations:** ^1^SUNY Eye Research ConsortiumBuffalo, NY, USA; ^2^Program in Neuroscience, SUNY – Stony BrookStony Brook, NY, USA; ^3^Medical Scientist Training Program, SUNY – Stony BrookStony Brook, NY, USA; ^4^Department of Neurobiology and Behavior, SUNY – Stony BrookStony Brook, NY, USA

**Keywords:** synaptic plasticity, GABA, inhibitory neurons, sensory cortex, inhibition

## Abstract

The mammalian neocortex is composed of a variety of cell types organized in a highly interconnected circuit. GABAergic neurons account for only about 20% of cortical neurons. However, they show widespread connectivity and a high degree of diversity in morphology, location, electrophysiological properties and gene expression. In addition, distinct populations of inhibitory neurons have different sensory response properties, capacities for plasticity and sensitivities to changes in sensory experience. In this review we summarize experimental evidence regarding the properties of GABAergic neurons in primary sensory cortex. We will discuss how distinct GABAergic neurons and different forms of GABAergic inhibitory plasticity may contribute to shaping sensory cortical circuit activity and function.

## Introduction

The many varieties of synaptic plasticity provide a high degree of flexibility to neural circuits, allowing organisms to adapt to changing environments and learn from their experiences. In primary sensory cortex different forms of synaptic plasticity can be induced by manipulations of sensory drive, some of which are limited to specific developmental windows (Hubel and Wiesel, 1970; Carmignoto and Vicini, 1992; Finnerty et al., 1999; Morales et al., 2002; Hensch, 2004; Maffei and Turrigiano, 2008a; Espinosa and Stryker, 2012; Levelt and Hübener, 2012). While most studies of sensory cortical plasticity have focused on the role of glutamatergic synapses, it is now clear that GABAergic inputs and their plasticity play a fundamental role in cortical circuit refinement throughout life.

GABAergic neurons comprise a variety of cell types with distinct physiological characteristics, anatomical locations and capacities for plasticity (Markram et al., 2004; Ascoli et al., 2008; Caspary et al., 2008; Maffei, 2011; Méndez and Bacci, 2011; Rudy et al., 2011; Vitalis and Rossier, 2011; Taniguchi, 2014). This diversity may be one of the most important features of cortical circuits, as it confers a wide range of possibilities for stabilizing, refining the activity of and setting the state of other neurons and neural circuits (Földy et al., 2004; Santhakumar and Soltesz, 2004). Tools to facilitate the direct investigation of these diverse neurons have only recently become available; therefore, our understanding of the role of GABAergic neurons in cortical circuit function is just beginning.

In this review we will discuss recent findings on the role of GABAergic synaptic transmission and plasticity in sensory cortex. We will focus on the role of local inhibitory circuits; however, it should be noted that long range projecting inhibitory neurons have been identified in neocortex (Germroth et al., 1989). These neurons have only recently begun to be functionally characterized (Melzer et al., 2012), suggesting that much work still needs to be done to fully understand the diversity of connectivity and action of GABAergic neurons in the brain. GABAergic interneurons are involved in sharpening sensory responses and regulating circuit excitability, and they are a *sine qua non* of experience-dependent circuit refinement (Ben-Ari et al., 2004; Heimel et al., 2011; Isaacson and Scanziani, 2011; Maffei, 2011; Levelt and Hübener, 2012; Le Magueresse and Monyer, 2013). We will provide evidence for these functions and discuss possible mechanisms involved, as well as common and unique features of GABAergic synaptic plasticity in different sensory cortices. We will conclude with an attempt to reconcile seemingly discrepant experimental results and suggest issues that in our opinion need to be addressed to push this field forward.

## GABAergic inhibition and computation in sensory cortex

The contribution of inhibition to neural network computation goes beyond that of only a regulator of circuit excitability. Inhibitory neurons form highly interconnected networks of electrically and synaptically coupled neurons, and they have a wide range of anatomical and physiological properties ideally suited for driving broad network synchronization (Kawaguchi and Kubota, 1997; Tamás et al., 1998; Galarreta and Hestrin, 1999; Gibson et al., 1999; Amitai, 2001; Galarreta and Hestrin, 2002; Pfeffer et al., 2013; Taniguchi, 2014). Many individual GABAergic neurons connect broadly to local excitatory neurons and specifically to local inhibitory neurons, allowing them to exert their influence over large portions of neural circuits (Hestrin and Galarreta, 2005; Oswald et al., 2009; Packer and Yuste, 2011; Fino et al., 2013; Pfeffer et al., 2013). GABAergic inhibitory neurons are, therefore, ideally situated to contribute to the generation of activity associated with wakefulness and cognitive processing (Tamás et al., 2000; Whittington et al., 2000; Oswald et al., 2009).

Inhibitory and excitatory inputs interact dynamically to maintain neural networks in a balanced state that favors neural computations (McCormick, 2002; Haider and McCormick, 2009). They are dynamically coordinated and co-activated during both spontaneous and sensory evoked activity both in acute slice preparations (Adesnik and Scanziani, 2010; Graupner and Reyes, 2013) and *in vivo* (Okun and Lampl, 2008; Adesnik and Scanziani, 2010). The coordination of excitatory and inhibitory inputs is believed to underlie dynamic modifications of functional cortical connectivity, and these rapid changes in functional connectivity may be necessary for sculpting sensory responses (Haider et al., 2007; Haider and McCormick, 2009). According to computational models, coordinated and balanced excitation and inhibition can promote decorrelated network activity, which would favor efficient information processing (Renart et al., 2010).

## Diversity of inhibitory neurons and their connectivity

Computational models and theories of cortical function often treat inhibitory neurons as a single functional class. However, cortical circuits contain groups of GABAergic neurons that can be distinguished according to their functional properties (Markram et al., 2004; Rudy et al., 2011; Taniguchi, 2014). The heterogeneity of GABAergic neuron subtypes has long hindered our understanding of inhibitory circuits and their specific roles in different aspects of circuit function; therefore, principles for identifying and naming the distinct populations of inhibitory neurons across species are being discussed to facilitate comparisons of results obtained from different species and experimental approaches (Ascoli et al., 2008; DeFelipe et al., 2013). One of the most important features of interneurons is their axonal morphology, which can be used to determine the primary subcellular compartment (axons, dendrites, or soma) that they target for inhibition (Somogyi et al., 1998).

In addition, differential gene expression has become a useful tool for identifying populations of interneurons. Nearly all sensory cortical GABAergic neurons are thought to detectably express one, and only one, of three proteins: the calcium binding protein parvalbumin (PV), the peptide hormone somatostatin (SOM), or the ionotropic serotonin receptor 5HT3aR (Kawaguchi and Kubota, 1997; Lee et al., 2010, 2013; Rudy et al., 2011). Expression of the peptide hormone vasoactive intestinal peptide (VIP) delineates a specific subpopulation of 5HT3aR positive interneurons (Lee et al., 2010; Rudy et al., 2011). Because these markers identify distinct populations and account for nearly all GABAergic neurons, they have provided a useful starting point for the functional investigation of groups of inhibitory neurons in sensory cortex (Rudy et al., 2011); however, there is some overlap in the expression of PV and SOM mRNA, and many more markers have been identified both immunohistochemically and through genetic screens that may help identify important functional subclasses (Cauli et al., 1997, 2000; Markram et al., 2004; Freund and Katona, 2007; Lee et al., 2010; DeFelipe et al., 2013; Taniguchi, 2014).

Interneurons have long been classified by their distinct morphological phenotypes and biophysical properties, and these correspond reasonably well with genetic markers. However, the evidence upon which these classifications are drawn relies on observations made across several species, brain regions and ages. These classifications represent the most common features of interneurons marked by these genes that have been observed, but the heterogeneity within these groups has not been systematically studied, especially across development, cortical layers and cortical regions. Most PV expressing neurons show fast spiking (FS) patterns when depolarized and can be classified morphologically as either basket cells or chandelier cells (Kawaguchi and Kubota, 1993; Wang et al., 2002; Markram et al., 2004; Xu and Callaway, 2009; Rudy et al., 2011). Basket cells have axons that synapse predominantly onto the soma and the perisomatic regions of cortical pyramidal neurons, wrapping around the soma like a basket (Kawaguchi and Kubota, 1997; Tamás et al., 2000). Chandelier cells, with axonal boutons resembling candlesticks or “cartridges” (Szentágothai and Arbib, 1974), are a population of interneurons whose axons predominately contact axon initial segments (Somogyi, 1977). Chandelier cells were originally thought to comprise a homogeneous subpopulation of FS PV positive neurons; however, recent evidence that many chandelier cells do not express PV suggests that two types of functionally distinct chandelier cells could exist (Taniguchi et al., 2013; Taniguchi, 2014). While most PV neurons are FS, depending upon the recording and classification method considered, not all PV neurons are FS and not all FS neurons are PV positive (Gray and McCormick, 1996; Cauli et al., 1997; Markram et al., 2004; Ma et al., 2006; Moore and Wehr, 2013; Taniguchi et al., 2013; but see Rudy et al., 2011).

The largest subgroup of SOM expressing neurons is Martinotti cells, which have regular spiking (RS) action potential widths when depolarized, and vertical axons that preferentially target superficial dendritic tufts (Kawaguchi and Kubota, 1997; Thomson et al., 2002; Wang et al., 2004; Ali and Thomson, 2008). However, SOM expressing synapses have also been observed on soma (Gonchar et al., 2002). In addition, a subgroup of SOM positive neurons has spike widths characteristic of FS cells and extensive axonal arborization within layer 4 (Ma et al., 2006; Miyoshi et al., 2007; Xu et al., 2013). The morphology and subcellular targeting of these neurons has not been fully characterized.

VIP is expressed in cells with morphologies consistent with both dendrite and soma targeting interneurons (Lorén et al., 1979; Morrison et al., 1984; Kawaguchi and Kubota, 1996, 1997; Lee et al., 2010; Miyoshi et al., 2010). 5HTR3aR/VIP- neurons comprise the majority of layer 1 neurons and neurogliaform cells (Lee et al., 2010). Neurogliaform cells form gap junction connections with many other classes of cortical interneurons (Simon et al., 2005). They have been proposed to use primarily volume rather than synaptic transmission of GABA (Oláh et al., 2009); however, they have the capacity to regulate specific portions of neural circuits (Chittajallu et al., 2013).

Beyond preference for targeting specific subcellular compartments, patterns of connectivity within and between interneuron subtypes and pyramidal neurons are beginning to emerge. For example, while PV and SOM positive neurons frequently contact pyramidal neurons, VIP positive neurons rarely inhibit pyramidal neurons (Pfeffer et al., 2013). Instead, VIP neurons predominately inhibit SOM neurons (Lee et al., 2013; Pfeffer et al., 2013; Pi et al., 2013), which in turn target VIP and PV neurons (Pfeffer et al., 2013). This connectivity scheme places VIP neurons in an ideal position to mediate disinhibition of excitatory neurons (Lee et al., 2013; Pi et al., 2013). In the primary input layer of sensory cortex, layer 4, basket cells receive direct thalamic input (Beierlein et al., 2003; Cruikshank et al., 2007) and can therefore shape cortical circuit activation through feedforward control over excitatory neuron activity (Gabernet et al., 2005). In addition to inhibiting excitatory neurons, many PV positive neurons target other PV positive neurons (Galarreta and Hestrin, 1999, 2002; Pfeffer et al., 2013).

The ability to perform experiments *ex vivo* in rodent models has allowed for high resolution analysis of biophysical properties and local connectivity of different populations of inhibitory neurons. For example, it was recently shown that even within the PV positive population of inhibitory neurons in primary visual cortex (V1), distinct biophysical properties (Helm et al., 2013) correlate with the level of expression of the SAP97 scaffold protein (Akgul and Wollmuth, 2013). Since inhibitory neuron populations with distinct biophysical properties, molecular markers, birth dates and birth regions have been identified, it has become possible to label specific subpopulations using genetic approaches to facilitate identification and recording from these groups of inhibitory neurons both *ex vivo* and *in vivo* (Taniguchi et al., 2011; Taniguchi, 2014).

While genetic approaches to marking neurons in intact animals are extremely powerful and promising, some of the strategies to genetically label interneuron populations may interfere with GABAergic transmission (Tamamaki et al., 2003; Wang et al., 2009; see discussion of the GAD67-GFP (Δneo) mouse line below) or mislabel neurons, potentially leading to confounds in the interpretation of the data, or producing contrasting findings (Hu et al., 2013). For example, neurons expressing Cre-dependent genes via the somatostatin promoter (Taniguchi et al., 2011) include a population (up to 5–10% of labeled neurons) of FS PV neurons (Hu et al., 2013; Pfeffer et al., 2013), while this degree of overlap has not been observed using immunohistochemical staining (Lee et al., 2010). In addition, the level of expression of neuronal markers used to identify inhibitory neurons can change during development (Wahle, 1993; Felmy and Schneggenburger, 2004) and/or in an activity-dependent fashion (Gomes da Silva et al., 2010; Martin del Campo et al., 2012; Hou and Yu, 2013), possibly affecting the classification of inhibitory neurons. Similarly, the subcellular compartments targeted by inhibitory synapses are under genetic control and can be modified in an activity dependent manner (Bloodgood et al., 2013). Finally, some functionally distinct population of inhibitory neurons may be distinguishable only by graded levels of gene expression, making them difficult to manipulate with genetically encoded tools alone. Comparing the properties, connectivity and function of inhibitory neurons in different circuits, species and developmental stages will be necessary to distinguish general properties of inhibitory neuron function from specific properties that depend on interneuron subtype, species, region or experimental manipulation. However, the heterogeneity within marked populations and potential plasticity of markers and subcellular targets must be taken into account.

## Manipulating and measuring the activity of GABAergic neurons *in vivo*

Several approaches have been employed to study the activity of GABAergic neurons *in vivo* (Table 1). The characteristic narrow spike waveform of FS inhibitory neurons allows them to be identified and separated from RS neurons, which are mostly pyramidal cells, in extracellular and juxtacellular recordings. Because identification of waveforms extracellularly is prone to error (Henze et al., 2000; González-Burgos et al., 2005; Gold et al., 2006), channelrhodopsins (ChR) can be expressed in subsets of interneurons and photoactivated to rapidly induce spikes and more precisely identify units recorded extracellularly (Cardin, 2012; Moore and Wehr, 2013). Multielectrode recording arrays are useful for recording extracellularly from several neurons simultaneously, but many are limited in their ability to record from superficial layers. Whole cell recording with *post-hoc* morphological reconstruction allows for more precise laminar identification than is possible with extracellular recordings, and depolarization induced firing patterns and membrane potential fluctuations can be measured. However, due to the size of interneurons relative to pyramidal neurons and their relative scarcity, this technique has low yield. To improve yield and specificity, recordings can be performed in transgenic animals expressing fluorescent markers in populations of GABAergic neurons. Using two-photon microscopy, patch pipettes can be targeted to neurons either expressing or lacking a specific marker (Liu et al., 2009; Gentet et al., 2010). Florescent labeling of GABAergic neurons can also be combined with calcium imaging, which greatly increases the number of neurons that can be simultaneously recorded from at the expense of resolving precise numbers of action potentials (Sohya et al., 2007; Kerlin et al., 2010). Unfortunately, techniques relying on transgenic animals and two photon microscopy are largely limited to studying the superficial layers of mouse cortex at present.

**Table 1:** **Approaches for recording the activity of inhibitory neurons and inhibitory postsynaptic responses *in vivo***.

**Technique**	**Cell type identification**	**Acquirable data**	**Limitations**	**Selected references**
Extracellular recordings	Spike width (FS vs. RS)	Spikes	Prone to misidentification, some arrays limited to deep layers	Simons, 1978; Swadlow and Weyand, 1987; Henze et al., 2000; González-Burgos et al., 2005; Gold et al., 2006
	Optogenetics	Spikes	Some arrays limited to deep layers	Cardin, 2012; Moore and Wehr, 2013
Juxtacellular recordings	Spike width (FS vs. RS)	Spikes	Low yield, cannot identify RS interneurons	Wu et al., 2008; Liu et al., 2009
	Two-photon guided targeting	Spikes	Superficial layers	Liu et al., 2009; Ma et al., 2010; Runyan et al., 2010
	Reconstruction	Spikes	Low yield	Wu et al., 2008; Ma et al., 2010; Runyan et al., 2010
Whole cell patch of interneurons	Reconstruction, depolarization induced firing patterns	Membrane potential, spikes	Very low yield	Azouz et al., 1997; Zhu and Connors, 1999; Hirsch et al., 2003; Monier et al., 2003
	Two-photon guided targeting	Membrane potential, spikes	Superficial layers, low yield	Gentet et al., 2010, 2012
Calcium dye imaging	Genetic labeling	Changes in calcium concentration	Superficial layers, cannot resolve individual spikes	Sohya et al., 2007; Kerlin et al., 2010
Whole cell patch of pyramidal neurons	Reconstruction, depolarization induced firing patterns	Somatic inhibitory/excitatory conductances, response reversal potentials	May not resolve dendritic inhibition	Anderson et al., 2000; Martinez et al., 2002; Crochet et al., 2011

*FS, fast spiking width. RS, regular spiking width*.

Whole cell recordings can also be used to measure inhibitory drive postsynaptically. Somatic inhibitory and excitatory currents/conductances can be measured to investigate how inhibition contributes to shaping sensory responses (Anderson et al., 2000). The dynamic reversal potential of a sensory response can also be calculated, allowing inferences to be made about the contribution of excitatory and inhibitory receptors to the response (Crochet et al., 2011; Sachidhanandam et al., 2013). These techniques are limited, however, in that they may fail to fully resolve inputs to distal dendrites. Distal inhibitory inputs may effectively shunt excitatory inputs that would have led to regenerative activity (Gidon and Segev, 2012) without themselves being fully resolved. Functional GABA mediated inhibition has been observed that requires regenerative dendritic activity to be measurable (Palmer et al., 2012). Measuring inhibitory currents/conductances requires minimizing the contribution of voltage gated channels and would obscure their inhibition. Further, the analysis of inhibitory responses with this approach does not allow experimenters to selectively identify the contribution of distinct inhibitory neuron subgroups to inhibitory synaptic potentials (or currents) evoked by sensory stimuli.

Pharmacology, genetics, optogenetics and alterations in sensory experience have all been used to manipulate GABAergic neurotransmission *in vivo* (Table 2). Application of the potent GABA_A_ receptor agonist muscimol has been used to increase inhibition (Reiter and Stryker, 1988); however, the ubiquity of GABA_A_ receptors makes it difficult to interpret the results of these experiments as being due specifically to increased synaptic and extrasynaptic inhibition versus the secondary effect of dampening all cortical activity. Benzodiazepines allosterically enhance GABAergic neurotransmission at specific GABA_A_ receptors with benzodiazepine binding sites (Benke and Möhler, 2006; Gielen et al., 2012) and are therefore a more selective tool for probing inhibitory circuit function. Conversely, GABAergic transmission can be disrupted either by administering GABA receptor antagonists or by impairing GABA synthetic pathways. For example, chronic administration of the GAD antagonist 3-mercaptopropionic acid (3-MPA) lowers cortical GABA (Harauzov et al., 2010). Transgenic mice that have altered expression of proteins related to GABAergic neuron function can be used to study the effects of either developmental or induced changes in GABAergic function (Hanover et al., 1999; Huang et al., 1999; Tamamaki et al., 2003; Fagiolini et al., 2004; Wang et al., 2009; Palmer et al., 2012; Nahmani and Turrigiano, 2014). To study the roles of interneuron groups in sensory processes, the light gated ion channel/pumps ChR and halorhodopsin/archaerhodopsin (HaR/ArchR) can be specifically expressed to excite or inhibit a class of neurons upon photoactivation (Cardin, 2012). While results using photoactivation are certainly interesting, it is unclear how artificially evoked (Cardin, 2012), broad patterns of interneuron activation relate to their physiological activation by sensory stimuli. A bold approach to increasing the specificity of photoactivation is to use single cell electroporation to limit expression to a small number of cells (Cottam et al., 2013).

**Table 2:** **Tools for manipulating inhibition *in vivo***.

**Class**	**Tools**	**Examples**	**Advantages**	**Limitations**	**Selected references**
Pharmacology	GABA_A_ receptor agonists	Muscimol		Broadly acting, silences neurons	Reiter and Stryker, 1988
	GABA_A_ receptor allosteric enhancers	Benzodiazepines and zolpidem	Enhances GABAergic transmission directly	Broadly acting	Fagiolini and Hensch, 2000; Hensch and Stryker, 2004
	GABA_A_ receptor antagonists	Picrotoxin and bicuculline	Blocks GABAergic transmission directly	Broadly acting	Sillito, 1975; Kyriazi et al., 1996
	GABA synthesis inhibitors	3-MPA		Broadly acting	Harauzov et al., 2010
	Growth factors	IGF-1	Probe signaling cascades	Broadly acting, actions beyond inhibition	Maya-Vetencourt et al., 2012
Genetics	Overexpression	BDNF	Ubiquitous or targeted overexpression	Depend on expression system	Hanover et al., 1999; Huang et al., 1999
	Knockout	GAD65	Full, ubiquitous knockout	Present through development	Fagiolini and Hensch, 2000
	Conditional knockout/knockdown		Targeted	Depends on knockout/knockdown system	
Optogenetics	Photoactivation	ChR	Targets genetic cell classes, good temporal response	Non-physiological stimulation parameters, non-physiological neurotransmission	Atallah et al., 2012; Cardin, 2012; Lee et al., 2012; Wilson et al., 2012
	Photo-inhibition	HaR/ArchR	Targets genetic cell classes	Incomplete inhibition, slow kinetics	Atallah et al., 2012; Gentet et al., 2012; Lee et al., 2013
Sensory experience	Sensory deprivation	Dark rearing, hearing loss, whisker removal	Delays circuit development	Non-specific effects	Benevento et al., 1992; Chittajallu and Isaac, 2010
	Environmental enrichment	Exercise, sensory enrichment	Re-opens critical periods	Non-specific effects	Sale et al., 2007
	Sensory alterations	Monocular Lid suture, selective whisker removal, high frequency hearing loss	Critical period plasticity useful for modeling human disease	Induces complex changes at excitatory and inhibitory synapses	Hubel and Wiesel, 1970; Drew and Feldman, 2009; Yang et al., 2011

*3-MPA, 3-mercaptopropionic acid. ChR, channelrhodopsin. HaR, halorhodopsin. ArchR, archaerhodopsin*.

Alterations in sensory experience that induce changes in GABAergic circuitry can be studied *in vivo* to allow inferences to be made about the effects of the circuit change on sensory responses. These inferences are, of course, limited by the plethora of changes that may be induced in cortical and subcortical circuits: excitatory, inhibitory and neuromodulatory. In this review, we will consider changes in sensory experience in three categories. Sensory deprivation will refer to manipulations that lead to an overall reduction of sensory drive (e.g., binaural hearing loss, dark rearing, or trimming all whiskers on a vibrissal pad). This type of manipulation is typically initiated shortly after birth. Deprivation of one sensory modality can alter cortical circuit activity and responsiveness to sensory stimuli even in sensory areas that are not directly driven by the sensory organ that has been manipulated (Zheng et al., 2014). Environmental enrichment will refer adding objects to an animal's cage that provide multisensory stimulation (Sale et al., 2009). Sensory alteration will refer to manipulations that reduce, or fundamentally alter, a specific sensory input, usually asymmetrically, without eliminating it entirely (e.g., monocular lid suture, surgical strabismus, selective hearing ablation and selective whisker removal). When these manipulations are induced during sensitive periods in postnatal development, they often lead to diminished sensory function if later reversed (Hubel and Wiesel, 1970; Carmignoto and Vicini, 1992; Finnerty et al., 1999; Morales et al., 2002; Hensch, 2004; Maffei and Turrigiano, 2008a; Espinosa and Stryker, 2012; Levelt and Hübener, 2012).

## GABAergic neuron sensory response properties

The diversity of genetic, morphological and electrophysiological properties, as well as patterns of connectivity of GABAergic neurons, suggests that different cell types may have distinct functional properties. Studies of GABAergic neurons' receptive field properties have confirmed this hypothesis, yet sometimes with contrasting results. This is not too surprising, given that important functional differences exist between cortical layers and neuronal subtypes, and different recording techniques selectively favor certain layers and interneuron subtypes over others (Table 1). Additionally, the laminar distribution and cortical organization of neurons with different response properties differs strikingly between species. Cats have ocular dominance and orientation columns, while rodents and rabbits do not (Hubel and Wiesel, 1963; Tiao and Blakemore, 1976; Murphy and Berman, 1979; Grinvald et al., 1986; Métin et al., 1988; Girman et al., 1999; Schuett et al., 2002; Van Hooser et al., 2005). Cats, rats, and mice all have different laminar distributions of neurons with simple and complex visual receptive fields (Hubel and Wiesel, 1962; Kelly and Van Essen, 1974; Gilbert, 1977; Gilbert and Wiesel, 1979; Lin et al., 1979; Parnavelas et al., 1983; Niell and Stryker, 2008). These interspecies differences in the gross organization of neuronal response properties raise the possibility that some features of inhibitory neuron response properties may also be species specific.

In cat V1 there is evidence in favor of FS inhibitory neurons being both broadly and narrowly tuned to sensory stimuli (Azouz et al., 1997; Hirsch et al., 2003; Cardin et al., 2007; Nowak et al., 2008). Tuning appears to vary by cortical layer, as FS neurons in the primary thalamorecipient layer, layer 4, are either broadly tuned or untuned (Hirsch et al., 2003; Cardin et al., 2007; Nowak et al., 2008). FS neurons outside layer 4 show tuning similar to RS neurons (Cardin et al., 2007), but are more binocularly driven (Aton et al., 2013). This suggests that inhibition may modulate sensory response properties in a lamina-specific fashion. In awake rabbits, putative layer 4 FS neurons have broader orientation, direction and frequency tuning, are more binocular and are more sensitive to contrast than putative RS neurons (Swadlow and Weyand, 1987; Swadlow, 1988; Zhuang et al., 2013).

In the anesthetized mouse, populations of interneurons identified as either PV positive or FS are more broadly tuned for orientation than both excitatory neurons (Niell and Stryker, 2008; Kuhlman et al., 2011; Zariwala et al., 2011; Atallah et al., 2012; Li et al., 2012b; Cottam et al., 2013; Runyan and Sur, 2013; but see Runyan et al., 2010) and SOM interneurons (Ma et al., 2010; Cottam et al., 2013). FS neurons are also more binocularly responsive than RS neurons (Yazaki-Sugiyama et al., 2009; Kameyama et al., 2010). Within the PV population, however, there is great tuning diversity, with some PV neurons being well tuned (Runyan et al., 2010; Zariwala et al., 2011; Runyan and Sur, 2013). Interestingly, PV neurons with longer dendrites have broader orientation selectivity than those with shorter dendrites (Runyan and Sur, 2013). Some of the heterogeneous aspects of PV neuron tuning may be due to the diversity of morphological groups of neurons expressing PV: basket cells (small and large) and a subgroup of chandelier cells. While both basket and chandelier cells exert powerful control over pyramidal neuron excitability and firing, they receive distinct sets of inputs, at least in somatosensory cortex (Xu and Callaway, 2009). SOM neurons exhibit weaker responses to visual stimuli than either excitatory or PV neurons (Ma et al., 2010) and exhibit sharper population orientation tuning than PV neurons (Ma et al., 2010; Cottam et al., 2013). Using optogenetic photoactivation of a small number of SOM neurons, it was shown that during visual stimulation, SOM neurons are able to more strongly inhibit responses of PV neurons than excitatory neurons. Interestingly, SOM neuron photoactivation sharpened PV neuron tuning (Cottam et al., 2013).

In layer 2/3 of GAD67-GFP (Δneo) mice, GABAergic neurons are only weakly orientation selective (Sohya et al., 2007; Bonin et al., 2011), and have similar binocularity to glutamatergic neurons (Gandhi et al., 2008). In these mice no difference in selectivity exists between FS and RS GABAergic neurons (Liu et al., 2009) or between SOM, PV, or VIP positive neurons (Kerlin et al., 2010). However, GAD67-GFP (Δneo) mice have reduced orientation selectivity of both GABAergic (Runyan et al., 2010) and glutamatergic neurons compared with control strains (Hagihara and Ohki, 2013; but see Liu et al., 2009). GAD67-GFP (Δneo) mice have decreased expression of GAD67 (Wang et al., 2009). Furthermore, their levels of GABA are reduced neonatally and in the adult (Tamamaki et al., 2003). These caveats should be carefully considered when interpreting data obtained using this strain.

In primary auditory cortex (A1) of anesthetized cats, putative FS inhibitory neurons have short response latencies and broad spectral tuning (Atencio and Schreiner, 2008). Similarly, rat layer 4 FS neurons have faster response latencies and broader tuning than layer 4 pyramidal neurons (Wu et al., 2008). In mice, units identified extracellularly as FS have faster response latencies and broader tuning than RS units (Moore and Wehr, 2013). Once subdivided into PV positive and PV negative units, however, PV positive units did not differ in frequency tuning or response latency from PV negative units, but did have higher spontaneous and evoked firing rates. They also had shallower response gain and were less well tuned for stimulus intensity than PV negative units. Very narrowly tuned PV positive neurons were found, but very rarely. These findings were interpreted as evidence for a role of PV neurons in modulating gain and intensity tuning (Moore and Wehr, 2013). Differently, in superficial layers PV neurons were found to be more broadly tuned than either excitatory or SOM positive neurons, while SOM neurons showed delayed responses compared to PV and excitatory neurons (Li et al., 2014b). VIP neurons are also tone responsive; however, their tuning properties have not been characterized (Pi et al., 2013).

In the barrel portion of primary somatosensory cortex (S1) in rats (paralyzed, anesthetized or sedated) and awake rabbits, putative FS inhibitory neurons show broader sensitivity to the direction of whisker deflection and/or to the number of whiskers stimulated than RS units (Simons, 1978; Simons and Carvell, 1989; Swadlow, 1989; Bruno and Simons, 2002; Gabernet et al., 2005; but see Zhu and Connors, 1999). In addition, they show faster and more reliable responses to stimulation than putative excitatory neurons, especially in layer 4 (Simons, 1978; Swadlow, 1989; Armstrong-James et al., 1993; Bruno and Simons, 2002; Swadlow and Gusev, 2002), where both excitatory and inhibitory neurons are driven monosynaptically from the thalamus (Beierlein et al., 2003; Cruikshank et al., 2007). Within rat layer 2/3, chandelier cells have broader receptive fields than other FS interneurons (Zhu et al., 2004). Within layer 1, deep-projecting GABAergic neurons are well tuned and respond to stimuli rapidly, whereas local interneurons respond slowly and are poorly tuned (Zhu and Zhu, 2004). In the hindpaw region of S1, a sizable portion of inhibitory neurons respond to ipsilateral stimulation (Palmer et al., 2012). Differently from rats and rabbits, putative FS inhibitory neurons in layer 4 of mouse barrel cortex show similar angular tuning to RS units (Kwegyir-Afful et al., 2013). In contrast to other cell types in mouse layer 2/3, SOM neurons are active during quite wakefulness and inhibited during sensation (Gentet et al., 2012). The inhibition of SOM neurons in S1 during whisking is dependent upon VIP neurons, which are driven to increase their activity during whisking by primary motor cortex (Lee et al., 2013).

Few studies have examined the tuning of GABAergic inhibitory interneurons in primary chemosensory cortices. In the primary gustatory cortex (GC) of anesthetized rats, putative FS inhibitory neurons are primarily tuned to one or two (of four) tastants, almost always including NaCl; while RS units are more broadly tuned and may respond several tastants (Yokota et al., 2011). In the primary olfactory cortex (Pir) of anesthetized rats, layer 1 GABAergic neurons are broadly tuned to odors compared to layer 2/3 pyramidal neurons (Poo and Isaacson, 2009).

Finally, recent studies have identified putative inhibitory interneuron involvement in multisensory integration and learning. Inhibitory neurons in layer 2/3 of a mouse visuotactile area between visual and somatosensory cortices exhibit less multisensory enhancement of evoked responses than pyramidal neurons, and their activation suppresses multisensory enhancement of activity in pyramidal neurons (Olcese et al., 2013). In mouse V1, a local inhibitory circuit can be driven by an auditory stimulus (Iurilli et al., 2012), and in GC of awake rats, a few putative FS inhibitory neurons were found that respond to auditory cues that triggered expectation of taste delivery (Samuelsen et al., 2012). Mouse A1 layer 2/3 VIP neurons and layer 1 interneurons inhibit other GABAergic neurons during auditory fear conditioning (Letzkus et al., 2011) and discrimination tasks (Pi et al., 2013), respectively.

While it is clear that there are lamina and species specific interneuron response properties, some common features exist within cell types (Table 3). FS and PV neurons, which predominantly provide somatic, perisomatic and axonal inhibition, tend to be broadly and bilaterally tuned, especially within layer 4. Differently, SOM neurons, which predominantly provide dendritic inhibition, tend to be less strongly responsive to, or inhibited by, stimuli, and those that are responsive to stimuli are tuned intermediately compared to PV and pyramidal neurons. It is unclear how FS SOM neurons and RS PV neurons fit into this scheme. Little is known about the tuning properties of 5HT3aR neurons, whether VIP positive or negative; however, the VIP positive subgroup has been implicated in mediating disinhibition during associative learning and active sensation (Lee et al., 2013; Pi et al., 2013). When comparisons have been made between subgroups of these populations, differences in response properties tied to morphology have been observed. Full understanding of the roles of GABAergic neurons, therefore, will require more detailed investigations of these subclasses, as well as further investigation into the function of genetically identified interneuron classes in subgranular layers.

**Table 3:** **Tuning width of inhibitory neurons by cell type**.

**Interneuron class**	**Sensory cortex**	**Layers**	**Tuning**	**Species**	**References**
FS	V1	4	Broadly tuned	Cat, Rabbit, Mouse	Swadlow and Weyand, 1987; Swadlow, 1988; Cardin et al., 2007; Li et al., 2012b; Zhuang et al., 2013
		4	Mixed	Cat	Hirsch et al., 2003
		2/3, 5/6	Well-tuned	Cat	Cardin et al., 2007
		Mixed	Broadly tuned	Cat, Mouse	Niell and Stryker, 2008; Nowak et al., 2008
		Mixed	Mixed	Cat	Azouz et al., 1997
	A1	4, Mixed	Broadly tuned	Cat, Rat, Mouse	Atencio and Schreiner, 2008; Wu et al., 2008; Moore and Wehr, 2013
	S1	4, Mixed	Broadly tuned	Rat, Rabbit	Simons, 1978; Simons and Carvell, 1989; Swadlow, 1989; Bruno and Simons, 2002; Gabernet et al., 2005
		4, Mixed	Well-tuned	Rat, Mouse	Zhu and Connors, 1999; Kwegyir-Afful et al., 2013
	GC	Mixed	Well-tuned	Rat	Yokota et al., 2011
PV	V1	2/3, 4	Broadly tuned	Mouse	Ma et al., 2010; Kuhlman et al., 2011; Zariwala et al., 2011; Atallah et al., 2012; Cottam et al., 2013; Runyan and Sur, 2013
		2/3	Mixed	Mouse	Runyan et al., 2010; Zariwala et al., 2011; Runyan and Sur, 2013
	A1	2/3	Broadly tuned	Mouse	Li et al., 2014b
		Mixed	Well-tuned	Mouse	Moore and Wehr, 2013
Chandelier	S1	2/3	Broadly tuned	Rat	Zhu et al., 2004
SOM	V1	2/3, 4	Well-tuned	Mouse	Ma et al., 2010; Cottam et al., 2013
	A1	2/3	Well-tuned	Mouse	Li et al., 2014b
VIP	A1		Tone responsive, tuning unknown	Mouse	Pi et al., 2013
Layer 1	S1	1: deep projecting	Well-tuned	Rat	Zhu and Zhu, 2004
	S1	1: local	Broadly tuned	Rat	Zhu and Zhu, 2004
	Pir	1	Broadly tuned	Rat	Poo and Isaacson, 2009

*FS, fast spiking width; V1, primary visual cortex; A1, primary auditory cortex; S1, primary somatosensory cortex; GC, primary gustatory cortex; Pir, primary olfactory cortex; PV, parvalbumin positive neurons; SOM, somatostatin positive neurons; VIP, vasoactive intestinal peptide positive neurons*.

## GABAergic inhibition and sensory responses in excitatory cells

The varied response properties of GABAergic neurons make them ideal candidates to modulate the activity of excitatory neurons during sensory processing. *In vivo* pharmacological manipulation of GABAergic transmission alters receptive fields properties in a number of different species and cortical regions, suggesting a role for cortical inhibition in controlling the stimulus preference and receptive field structure of excitatory neurons (Sillito, 1975, 1979; Sillito et al., 1980; Kyriazi et al., 1996; Tremere et al., 2001; Li et al., 2002; Foeller et al., 2005; Jeong et al., 2009; Razak and Fuzessery, 2009; Isaacson and Scanziani, 2011; Katzner et al., 2011). However, the results of grossly disrupting or augmenting inhibition in cortex do not provide evidence that GABAergic neurons dynamically shape receptive fields, and their contribution to shaping the receptive fields of glutamatergic neurons is hotly debated (Isaacson and Scanziani, 2011; Liu et al., 2011; Wu et al., 2011; Priebe and Ferster, 2012). Depending on the location and specificity of inhibitory inputs onto a pyramidal cell, GABAergic neurons with a range of tuning properties could contribute inhibition tuned similar to, more tightly than or more broadly than excitatory inputs to that neuron, or tonic, stimulus invariant inhibition.

There is general agreement that an inhibitory mechanism contributes to pyramidal neurons having narrower spiking receptive fields than subthreshold receptive fields (Priebe and Ferster, 2008; Isaacson and Scanziani, 2011; Tan et al., 2011; Wu et al., 2011). This sharpening effect of inhibition occurs, at least in part, through an “iceberg” effect, where inhibition contributes to the spike threshold, below which changes in membrane potential do not alter firing. This effect can occur independently of the relative tuning widths of inhibitory and excitatory inputs (see Isaacson and Scanziani, 2011). Static changes in the level of inhibition, independent of tuning, could alter the sharpness of the membrane potential dynamics in response to spiking input/output transformation, effecting a change in gain. Inhibitory inputs with different tuning than excitatory inputs, if they exist, would provide additional sharpening (Wu et al., 2008; Liu et al., 2011).

In V1, lateral inhibition could account for many receptive field properties, including orientation tuning, spatial segregation of light and dark receptive subfields, direction selectivity, contrast invariance and suppression. However, it has also been proposed that most of these response properties can be accounted for by a model based on the properties of feedforward, excitatory thalamic inputs that have been observed *in vivo*, coupled with a spike threshold determined in part by inhibitory inputs (Priebe and Ferster, 2012). However, the ability to account for most receptive field properties without lateral inhibition does not rule out a role for lateral inhibition. We will focus our discussion on the evidence for and against lateral inhibition being observed and playing a role in shaping receptive fields.

Inhibitory and excitatory light and dark receptive subfields are largely spatially segregated in the visual cortex of cats (Ferster, 1988; Hirsch et al., 1998; Priebe and Ferster, 2005). The spatial opposition of inhibition and excitation was proposed to allow for linear summation of inputs: at a given location within a receptive field, dark and light stimuli evoke opposite responses (Priebe and Ferster, 2005). Conversely, voltage clamp recordings from simple cells in mice have revealed spatial overlapping of receptive subfields, with inhibitory light and dark subfields similarly centered and lying between slightly segregated excitatory light and dark subfields. This suggests a role for inhibition in the segregation of light and dark receptive fields in mice (Liu et al., 2010).

Orientation tuning of excitatory and inhibitory inputs to pyramidal neurons has been observed to be either similarly tuned (Anderson et al., 2000; Tan et al., 2011) or to have a variety of tuning schemes (Martinez et al., 2002; Monier et al., 2003) in cat V1. Orientation tunings of inhibition and excitation were found to be similar in layers 2–4 but different in layer 5 (Martinez et al., 2002). In mice, similar tuning (Tan et al., 2011) and tuning of inhibition just broader than excitation (Liu et al., 2011; Li et al., 2012b) have both been reported. To address whether specific subclasses of inhibitory neurons have the capacity to alter orientation tuning, optogenetic stimulation has been used to control the activation of GABAergic neurons during visual stimulation while recording responses in superficial excitatory neurons. Photoactivation of PV neurons preferentially inhibited responses to optimal orientation without changing orientation selectivity (Atallah et al., 2012; Wilson et al., 2012), while photoactivation of SOM neurons inhibited responses at all orientations equally, sharpening orientation selectivity (Wilson et al., 2012). However, in a contrasting study PV neuron photoactivation sharpened orientation selectivity with minimal effects at optimal orientations, while SOM and VIP positive neurons' photoactivation suppressed firing but did not alter orientation tuning (Lee et al., 2012). Further, performance in an orientation discrimination task was improved by selective photoactivation of PV positive neurons (Lee et al., 2012). The differences in results may be due to the stimulation parameters used (Lee et al., 2012). As discussed above, it is unclear how these results relate to physiological roles of inhibitory neurons.

Experiments examining the role of inhibition in sharpening tuning width in sensory areas other than V1 have yielded similarly contrasting results. In rodent A1, frequency tuning of inhibitory inputs has been found to be approximately similar to (Wehr and Zador, 2003; Zhang et al., 2003; Tan et al., 2004; Froemke et al., 2007; Tan and Wehr, 2009; Dorrn et al., 2010; Li et al., 2014a) or slightly broader than (Wu et al., 2008; Sun et al., 2010) excitatory inputs. In addition, intensity tuning of excitatory and inhibitory inputs to non-monotonically tuned neurons has been observed to be similarly (Wehr and Zador, 2003) and differentially tuned (Wu et al., 2006; Tan et al., 2007). In rat Pir, inhibitory inputs to layer 2/3 pyramidal neurons are broadly tuned to odors (Poo and Isaacson, 2009).

Experimental evidence in favor of a role for lateral inhibition in sharpening the selectivity of tuning curves has been found, but it has not been observed consistently either within species or cortical regions (Table 4; Priebe and Ferster, 2012). Possible reasons for these inconsistencies include anesthetic choices, stimulus parameters, age of animals, recording layers and experimental approaches. Recent data from layer 2/3 of mouse V1 demonstrated that in awake mice the ratio of inhibition to excitation observed in response to visual stimuli is much greater than under anesthesia (Haider et al., 2013). Further, strong inhibition could be evoked by stimulation in the receptive field surround only during wakefulness (Haider et al., 2013). Thus, the state of a neuron or network can profoundly affect receptive field properties and alter the function of inhibition within a circuit. In layer 4 of mouse V1, increasing the contrast of a grating stimulus broadens inhibitory tuning, leading to contrast-dependent sharpening of orientation selectivity (Li et al., 2012a). Age-dependent sharpening (Dorrn et al., 2010) and widening (Sun et al., 2010) of inhibitory tunings have been reported in rat A1. Developmental broadening of inhibition, both in the tuning of PV neurons and in inhibitory inputs to pyramidal cells has been observed in mouse V1 (Kuhlman et al., 2011; Li et al., 2012b). Therefore, there may be specific conditions and developmental windows in which lateral inhibition contributes substantially to sharpening neuronal receptive fields. A neural network computational model has been developed that can operate in both co-tuned and lateral inhibition modes, depending upon the input received (Levy and Reyes, 2011), and another set of theoretical models suggests that the diversity of receptive fields seen *in vivo* may be accounted for by the existence of multiple network architectures existing simultaneously (de la Rocha et al., 2008; Piëch et al., 2013). These results, coupled with the limitations of recording inhibition somatically, make definitive conclusions against a role for lateral inhibition in sharpening response selectivity tenuous.

**Table 4:** **Relative tuning of excitatory and inhibitory somatic conductances/currents in excitatory neurons**.

**Sensory cortex: stimulus parameter**	**Layers**	**Anesthesia**	**Age**	**Species**	**Inhibitory and excitatory tuning widths**	**References**
V1: Orientation	Mixed, 2/3, 4	Thiopental, ketamine/thiopental, diprivan/sufentanil citrate, pentobarbital/chlorprothixene	Adult	Cat, Mouse	Co-tuned	Anderson et al., 2000; Martinez et al., 2002; Tan et al., 2011
	4	Urethane/chlorprothixene	Pre-critical period	Mouse	Co-tuned	Li et al., 2012b
	Mixed	Alfaxolone/alfadolone,	Mixed	Cat	Mixed	Monier et al., 2003
	5	Ketamine/thiopental, diprivan/sufentanil citrate	Adult	Cat	Different excitatory and inhibitory tuning	Martinez et al., 2002
	2/3, 4	Urethane/chlorprothixene	Adult, critical period	Mouse	Inhibition broader than excitation	Liu et al., 2011; Li et al., 2012b
A1: Frequency	Mixed, 3–5	Ketamine/medetomidine, pentobarbital	Pre-critical period, critical period, adult	Rat, Mouse	Co-tuned/Similar	Wehr and Zador, 2003; Zhang et al., 2003; Tan et al., 2004; Froemke et al., 2007
	Mixed	Ketamine/xylazine	Pre-critical period	Rat	Different excitatory and inhibitory tuning	Dorrn et al., 2010
	Mixed	Ketamine/xylazine	Adult	Rat	Co-tuned	Dorrn et al., 2010
	4	Ketamine/xylazine	Pre-critical period	Rat	Co-tuned	Sun et al., 2010
	4	Ketamine/xylazine	Adult	Rat	Inhibition broader than excitation	Wu et al., 2008; Sun et al., 2010
A1: Intensity	Mixed	Ketamine/medetomidine	Pre-critical period/critical period	Rat	Co-tuned/Similar	Wehr and Zador, 2003
	Mixed, 4	Ketamine/xylazine, pentobarbital	Adult	Rat	Umbalanced	Wu et al., 2006; Tan et al., 2007
Pir: Odor	2/3	Urethane, ketamine	Pre-critical period, critical period	Rat	Inhibition broader than excitation	Poo and Isaacson, 2009

The relative timing of excitatory and inhibitory inputs to a neuron can influence its ability to integrate inputs. Inhibition that follows excitation with a brief delay can create a narrow temporal window within which spiking can occur (Wehr and Zador, 2003; Wilent and Contreras, 2004; Higley and Contreras, 2006). If the delay before the onset of inhibition varies with the stimulus, this delay could participate in coding. In rat S1, excitatory and inhibitory inputs to layer 3 and 4 neurons are poorly tuned for the direction of a whisker deflection; however, the temporal gap before the onset of inhibition is greatest for the preferred direction (Wilent and Contreras, 2005). In layer 6 of rat A1, a majority of pyramidal neurons receive strong tone-evoked synaptic inhibition prior to excitation and are thus inhibited by tonal stimulation (Zhou et al., 2010). Interestingly, the time course of excitatory and inhibitory responses to oriented gratings in V1 differs between species: inhibition and excitation wax and wane in opposition in cats (Hirsch et al., 1998; Anderson et al., 2000; Monier et al., 2003; Priebe and Ferster, 2005; Tan et al., 2011), while they are modulated synchronously in the mouse (Tan et al., 2011). Species-specific differences in light and dark receptive subfields have been proposed to underlie this discrepancy (Tan et al., 2011).

In addition to possibly mediating spike timing and tuning curves, inhibition may play a role in response suppression and in maintaining sparseness of spiking responses. Response suppression occurs when a stimulus that would not by itself alter firing of a neuron inhibits the response of that neuron to another stimulus. While GABAergic neurons mediate some forms of response suppression, they likely do not mediate all response suppression. For example, when two tones of the same frequency are played in rapid succession, the cortical response to the second tone is suppressed. As both the onset and offset of stimuli can evoke neuronal responses with transient inhibitory and excitatory components, one hypothesis explaining this effect involves the first tone's offset evoking inhibition that suppresses the response to the second tone (Borg-Graham et al., 1998; Wehr and Zador, 2005; Scholl et al., 2010). However, Scholl et al. found that in rat A1 there is different frequency tuning of inputs for tone onset and offset (Scholl et al., 2010). This differential tuning allows the onset and offset of a given tone to evoke different excitatory and inhibitory current ratios, albeit transiently. Therefore, “forward” suppression of a second tone of the same frequency can only be mediated through feedforward inhibition generated by the first tone's onset if the second tone follows within the brief time window of the first tone's onset inhibitory current (Scholl et al., 2010).

In V1, a stimulus placed adjacent to a neuron's spiking receptive field can alter its responses to stimuli within the spiking receptive field, a phenomenon referred to as surround suppression. Surround suppression has been shown to be mediated, at least in part, through intracortical inhibition (Ozeki et al., 2009; Haider et al., 2010; Adesnik et al., 2012). However, there may be multiple mechanisms through which surround suppression occurs, depending on the nature of the stimuli. For example, in cats, surround suppression by optimally oriented gratings decreases the total excitatory and inhibitory inputs onto a cell: a transient increase in inhibition can be evoked by surround stimuli, which ultimately leads to suppression of both cortical excitatory and inhibitory inputs (Ozeki et al., 2009). For “naturalistic” stimuli, movies of wildlife, adding stimuli to the receptive field surround increases spiking reliability and firing sparseness (Haider et al., 2010). This effect is mediated through an increase in evoked inhibitory potentials throughout the stimulus duration and corresponds with an increase in FS interneuron firing (Haider et al., 2010). Perhaps the discrepancies between these studies could be accounted for by the ever changing nature of the “naturalistic” stimuli, with the many moving edges evoking successive bouts of inhibition that blend together (Haider et al., 2010). In layer 2/3 of awake mice, inhibition through SOM positive interneurons accounts for approximately 30% of surround suppression by optimally oriented gratings (Adesnik et al., 2012).

In layer 5 of S1, stimulation of the ipsilateral paw just prior to stimulating the contralateral paw suppresses the neuronal response to the contralateral paw. This suppression is mediated by layer 1 interneurons driven by colossal projections that provide dendritic inhibition via GABA_B_ receptor activation (Pérez-Garci et al., 2006; Palmer et al., 2012). These findings suggest that multiple intracortical mechanisms exist to generate suppression, with different mechanisms likely contributing to the suppression of different types of stimuli.

Inhibition is ideally suited to contribute to the maintenance of response sparseness. Interestingly, while S1/barrel cortex layer 2/3 pyramidal neurons uniformly show membrane potential responses to whisker stimulation, only ~10% spike during active touch (Crochet et al., 2011). This has been attributed to whisker responses having reversal potentials below spike threshold (Crochet et al., 2011; Sachidhanandam et al., 2013). These responses have reversal potentials well below those of excitatory receptors, suggesting that GABA receptor mediated currents may contribute to preventing these neurons from reaching spike threshold (Sachidhanandam et al., 2013). In favor of this interpretation, there is experimental evidence that whisker stimulation activates inhibitory neurons (Simons, 1978; Simons and Carvell, 1989; Swadlow, 1989; Armstrong-James et al., 1993; Zhu and Connors, 1999; Bruno and Simons, 2002; Swadlow and Gusev, 2002; Beierlein et al., 2003; Zhu et al., 2004; Zhu and Zhu, 2004; Gabernet et al., 2005; Cruikshank et al., 2007; Kwegyir-Afful et al., 2013; Lee et al., 2013; Sachidhanandam et al., 2013) and increases inhibitory drive onto excitatory neurons (Wilent and Contreras, 2005). Optogenetic photoactivation of PV neurons during a detection task both masked subthreshold responses and prevented behavioral responses (Sachidhanandam et al., 2013). These results suggest that inhibition can be precisely tuned to allow sparse firing in layer 2/3, keeping most neurons just below spike threshold after stimulation.

Additional circuit precision can be gained through subcellular compartment specific inhibition. While S1 layer 5 pyramidal neurons are rarely connected to each other directly, they are more commonly coupled disynaptically through layer 5 Martinotti cells (Silberberg and Markram, 2007). When activated, these Martinotti cells can prevent the generation of calcium spikes in the dendritic tufts of layer 5 pyramidal neurons (Murayama et al., 2009). Modeling of this circuit suggests that Martinotti cells are responsible for maintaining the dynamic range of dendritic calcium responses to paw shock that are observed *in vivo* (Murayama et al., 2009).

GABAergic neurons do not only contact excitatory neurons, but form networks of electrically and synaptically coupled interneurons, often comprised of the same cell type, that can be activated synchronously (Hestrin and Galarreta, 2005; Oswald et al., 2009; Pfeffer et al., 2013). This connectivity is especially well suited to allow for synchronization of local circuits and is thought to contribute to the generation of oscillatory network behaviors (Tamás et al., 2000; Whittington et al., 2000; Oswald et al., 2009). The synchronicity of membrane potential oscillations between neurons can vary depending on stimuli. Neurons with similar receptive fields increase their membrane potential cross-correlation in response to optimal stimuli and decrease it in response to non-optimal stimuli (Lampl et al., 1999). Regardless of relative tuning, coherence in the gamma band between neurons increases for a wide variety of visual stimulus conditions (Yu and Ferster, 2010). Fast rhythmic bursting cells can fire bursts of action potentials in the gamma range (20–80 Hz) in response to visual stimuli, possibly contributing to visually evoked gamma oscillations (Cardin et al., 2005). This evoked gamma activity is dependent upon synaptic drive and is not generated only by the membrane properties of the chattering cells (Cardin et al., 2005). Gamma frequency oscillations measured in local field potentials can be evoked *in vivo* by optogenetic stimulation of PV positive neurons, suggesting that FS neurons may drive stimulus-evoked activity in the gamma band (Cardin et al., 2009). Photoactivation of these neurons to drive gamma oscillations during whisker deflection increases the precision of excitatory neuron responses and alters the amplitude and timing of responses in an oscillation phase dependent manner (Cardin et al., 2009).

Future work will be necessary to unravel how specific interneuron subtypes contribute to modulating sensory responses, fully understand the roles they play in higher order processing, and address whether there are specific conditions under which lateral inhibition sharpens receptive fields. One recent study in auditory cortex has begun to examine the cell type specific contributions of inhibition to modulating pyramidal cell activity during learning: VIP neurons were found to respond preferentially to reinforcement (Pi et al., 2013). SOM neurons were rapidly inhibited by VIP neurons, which caused an increase in firing of a subset of pyramidal neurons. This finding was interpreted to mean that activation of VIP neurons by reinforcement cues alters the gain of pyramidal neurons via disinhibition of SOM neurons (Pi et al., 2013). Together, the results presented in this section raise the intriguing possibility that the diversity in morphology and biophysical properties of GABAergic neurons is matched by their many functions.

## Development of GABAergic neurons and synapses in sensory cortex

GABAergic neurons and synapses undergo a process of postnatal maturation which extends for quite some time after birth and co-occurs with the development of many functional properties of excitatory neurons (Espinosa and Stryker, 2012). Here, we will focus on the developmental changes that occur after inhibitory neurons have reached their target location within the cortical mantle and after GABAergic synaptic transmission has transitioned from excitatory to inhibitory. Exhaustive readings of earlier developmental processes can be found elsewhere (see Cherubini et al., 1991; Xu et al., 2003; Li and Xu, 2008; Gelman and Marín, 2010; Rudy et al., 2011; Ben-Ari et al., 2012; Ciceri et al., 2013; Taniguchi, 2014).

The intrinsic properties of inhibitory neurons change during postnatal development. In rodent V1, PV FS neurons show a progressive decrease in membrane time constant, cell capacitance and action potential width and an increase in intrinsic excitability during the first 2 weeks after eye opening (Goldberg et al., 2011; Lazarus and Huang, 2011). Conversely, SOM positive neurons show a progressive increase in membrane time constant, input resistance and spike frequency adaptation (Lazarus and Huang, 2011). The properties of inhibitory synapses also change during this developmental time window (Morales et al., 2002; Heinen et al., 2004; Maffei and Turrigiano, 2008a; Pinto et al., 2010). During the postnatal weeks following eye opening there is a progressive increase in a1 containing GABA_A_ receptors and a progressive decrease in a3 and a5 containing GABA_A_ receptors. Significant changes also occur in the expression of γ, δ and ε subunits (Heinen et al., 2004). Changes in receptor subunit composition underlie changes in conductance, clustering and susceptibility to allosteric modulators: all factors that play fundamental roles in determining the amplitude and time course of postsynaptic events (Bosman et al., 2002; Heinen et al., 2004; Möhler, 2006; Eyre et al., 2012). In mature brains GABA_A_ receptors containing specific α subunits are primarily located in specific subcellular compartments: α1 and α3 containing receptors are enriched at synapses on the soma and perisomatic regions of pyramidal neurons (Klausberger et al., 2002), α2 containing receptors are preferentially located at axo-axonal synapses (Nusser et al., 1996), α5 containing receptors are preferentially located at dendritic synapses (Ali and Thomson, 2008), and α4 and α6 containing receptors predominantly mediate extrasynaptic inhibition (Wisden et al., 2002; Chandra et al., 2006; Wu et al., 2012). Whether GABA_A_ receptor subunits are similarly localized during development is not known; however, the observed changes in levels of expression suggest that changes in the subcellular targeting of GABAergic synapses may occur.

Comparable processes of protracted maturation of inhibitory synapses have been reported in A1 and S1. In both regions there is a progressive increase in spontaneous IPSC amplitude (Kobayashi et al., 2008; Zhang et al., 2011; Takesian et al., 2012), and in A1 there are many developmentally regulated changes in inhibitory neuron intrinsic properties and in evoked synaptic strength (Takesian et al., 2010, 2012, 2013; Oswald and Reyes, 2011; Kinnischtzke et al., 2012).

Developmental changes in inhibitory circuit function go beyond subunit composition, strength and kinetic properties. Inhibitory drive tends to increase during postnatal development (Blue and Parnavelas, 1983; Morales et al., 2002; Chattopadhyaya et al., 2004; Maffei and Turrigiano, 2008a; Kuhlman et al., 2011; Li et al., 2012b). Changes in the strength of inhibitory inputs have been shown to decrease the ability of excitatory neurons to fire action potentials (Saraga et al., 2008; Pouille et al., 2013). Thus, developmental regulation of inhibitory synaptic strength may have significant effects on the activity of excitatory neurons. Precise patterns of neural activity are required for the induction of different forms of plasticity; therefore, changes in circuit activity may alter how stimuli drive changes at synapses. Patterns of activity or sensory experiences that facilitate the induction of long term potentiation (LTPi) or depression (LTDi) at inhibitory synapses could gate the induction of specific forms of plasticity at glutamatergic synapses in cortical circuits (Levelt and Hübener, 2012). Gating of excitatory plasticity could occur by altering activity patterns of glutamatergic neurons (Aton et al., 2013), by activating neuromodulatory signals (Huang et al., 2013) and/or through the activation of intracellular signaling cascades (Hayama et al., 2013; Wang and Maffei, 2014).

The capacity for plasticity at inhibitory synapses in V1 changes sharply during postnatal development at a time point corresponding to the transition between the pre-critical and the critical periods for visual cortical plasticity (Lefort et al., 2013). In response to the same pattern of activity, monosynaptic connections from FS neurons onto layer 4 pyramidal neurons show LTDi in the pre-critical period and LTPi in the critical period (Lefort et al., 2013). This shift in capacity for plasticity parallels that reported for layer 4 recurrent excitatory connections (Wang et al., 2012). Surprisingly, the switch in sign of plasticity at excitatory synapses can be reversed by inducing inhibitory plasticity in a connection specific fashion, indicating that inhibitory inputs may contribute to selective rewiring of local circuits despite their widespread connectivity (Wang and Maffei, 2014). This interaction between inhibitory and excitatory plasticity is mediated by G-protein signaling; therefore, it may affect the pyramidal neuron long after its induction (Wang and Maffei, 2014). Such a signaling crosstalk between mechanisms for excitatory and inhibitory plasticity could provide a memory trace that promotes experience-dependent rewiring of local microcircuits with a high degree of specificity and contributes to developmental circuit refinement.

During the developmental window in which inhibitory synapses mature, there are also substantial changes in glutamatergic receptor subunit composition, subcellular localization and function (Carmignoto and Vicini, 1992; Nase et al., 1999; Hensch, 2004; Corlew et al., 2007; Yashiro and Philpot, 2008), voltage signal propagation through the cortical circuit (Barkat et al., 2011; Griffen et al., 2013) and receptive field properties (Fagiolini et al., 1994; Katz and Shatz, 1996; Huang et al., 1999; Zhang et al., 2001; Inan and Crair, 2007; Wang et al., 2010; Espinosa and Stryker, 2012; Chen et al., 2014; Li et al., 2014a). Whether and how GABAergic neurotransmission contributes to these developmental processes is not fully understood. Evidence is beginning to emerge that changes in the receptive fields of the inhibitory drive onto excitatory neurons may at least partially underlie developmental receptive field sharpening (Dorrn et al., 2010; Sun et al., 2010; Li et al., 2012b). However, the underpinning synaptic and circuit mechanisms of this process remain elusive.

## Sensory deprivation impairs the development of GABAergic inhibition

The maturation of inhibitory synapses is strongly influenced by sensory experience. Visual deprivation induced by raising animals in the dark from birth or shortly thereafter disrupts visual response properties (Benevento et al., 1992; Fagiolini et al., 1994; Gianfranceschi et al., 2003; but see Rochefort et al., 2011) and delays the formation of mature innervation onto excitatory neurons; whereas dark rearing started after eye opening does not affect inhibitory drive (Morales et al., 2002; Maffei et al., 2010). Dark rearing decreases the level of expression of GAD65 in layer 2/3 inhibitory neurons (Kreczko et al., 2009) and prevents postnatal increases in IPSC amplitude onto pyramidal neurons from occurring, consistent with a delay in GABAergic input maturation (Morales et al., 2002). Interestingly, dark rearing mice blocks the developmental broadening of the orientation tuning of PV neurons, but it does not fully prevent the developmental sharpening of the orientation tuning of pyramidal neurons in layers 2–4 (Kuhlman et al., 2011; Rochefort et al., 2011; Li et al., 2012b), or of the inhibitory inputs to layer 4 pyramidal neurons (Li et al., 2012b). Dark rearing also delays the maturational increase in the coincidence of binocular inputs to pyramidal neurons in layers 2–4 (Chen et al., 2014). Pharmacologically enhancing inhibition with benzodiazepines early in development disrupts binocular matching of orientation preference in complex cells (Wang et al., 2010, 2013a), widens the spacing of orientation columns and disrupts direction selectivity (Hensch and Stryker, 2004). Together with sensory deprivation experiments, these results suggest that the normal development of GABAergic inhibition is essential for the proper maturation of visual cortex.

Auditory experience also plays a major role in the development of inhibitory inputs in A1, as early hearing loss, either conductive or sensorineural, alters GABAergic neurotransmission (Kotak et al., 2005, 2008; Takesian et al., 2010, 2012). Early conductive hearing loss delays the developmental decrease in IPSC decay time constant (Takesian et al., 2012), and produces a significant, long lasting decrease in spontaneous IPSC amplitude, suggesting the induction of long term depression of inhibitory drive (Takesian et al., 2012). Auditory experience is not only necessary for the maturation of inhibition in A1, but also regulates the capacity for plasticity at inhibitory synapses (Xu et al., 2010) and the feedforward thalamocortical drive onto different classes of inhibitory neurons (Takesian et al., 2013).

While whisker plucking prevents the maturation of feedforward inhibition onto excitatory neurons in the barrel cortex (Chittajallu and Isaac, 2010), the mechanisms engaged in driving GABAergic synapse maturation in S1 are unknown. Across cortices, early sensory deprivation delays the maturation of inhibitory synapses, but how experience-dependent, developmentally regulated cellular and synaptic changes in inhibition directly influence sensory responses is unclear.

## GABAergic maturation and critical period plasticity

Experiments with early sensory deprivation have shown that sensory experience is necessary for the maturation of inhibitory neurons and their synapses. However, if sensory manipulations are started later in postnatal development, for example during identified sensitive periods whose onset has been shown to correlate with the time course of the maturation of inhibition, sensory cortex is highly sensitive to even brief alterations in sensory drive (Hubel and Wiesel, 1970; Carmignoto and Vicini, 1992; Finnerty et al., 1999; Morales et al., 2002; Hensch, 2004; Maffei and Turrigiano, 2008a; Espinosa and Stryker, 2012; Levelt and Hübener, 2012). For example, monocular lid suture decreases the ratio of V1 neuronal spiking responses to the stimulation of the closed eye compared with the open eye, a phenomenon known as ocular dominance plasticity (Wiesel and Hubel, 1963). Ocular dominance plasticity is effectively induced by lid suture during a limited time window in postnatal development known as a critical period (Hubel and Wiesel, 1970; Fagiolini et al., 1994; Gordon and Stryker, 1996).

The maturation of inhibitory synapses has been proposed as a critical step for the modulation of the onset and duration of critical periods (Hensch et al., 1998). Manipulations of inhibition *in vivo* during early postnatal development shift the onset of the critical period for visual cortical plasticity in V1. In GAD65 knockout mice, which have low levels of GABA throughout life, the critical period never opens (Fagiolini and Hensch, 2000). Increasing intracortical inhibitory drive with benzodiazepines or zolpidem in early development, or at any time in GAD65 knockouts, leads to the onset of a window for ocular dominance plasticity (Fagiolini and Hensch, 2000; Fagiolini et al., 2004; Wang et al., 2013a; Chen et al., 2014). This effect is mediated by GABA_A_ receptors containing α1 subunits, which are preferentially located at FS to pyramidal neuron somatic synapses (Fagiolini and Hensch, 2000; Klausberger et al., 2002; Iwai et al., 2003; Fagiolini et al., 2004; Katagiri et al., 2007). Either overexpressing BDNF or removing polysialic acid accelerates the development of inhibitory synapses and causes a precocious critical period (Hanover et al., 1999; Huang et al., 1999; Di Cristo et al., 2007). Similarly, sensory deprivation by dark rearing prolongs the onset of sensitivity to ocular dominance plasticity until the animal is introduced to light (Cynader and Mitchell, 1980; Mower and Christen, 1985; Mower, 1991; Fagiolini et al., 1994).

Conversely, pharmacologically decreasing inhibition in adult animals can reopen a window for ocular dominance plasticity (Harauzov et al., 2010; Maya-Vetencourt et al., 2012). This can be accomplished by reducing cortical GABA with GAD inhibitors, with the peptide hormone IGF-1, or by blocking GABA_A_ receptors (Harauzov et al., 2010; Maya-Vetencourt et al., 2012). Environmental enrichment, which also decreases intracortical inhibition, can restore the capacity for ocular dominance plasticity in adult rodents in an IGF-1 dependent manner (Sale et al., 2007; Baroncelli et al., 2010; Maya-Vetencourt et al., 2012). Direct evidence that the age of inhibitory neurons, rather than overall inhibitory tone, plays a major role in critical period regulation comes from experiments where implants of GABAergic neural progenitors from embryonic mice were made into V1 of older mice. These implants later opened a window for ocular dominance plasticity that matched the age of the implanted neurons (Southwell et al., 2010). Substantial evidence exists that the maturation of inhibition regulates the timing of critical periods. Uniformly, manipulations that prevent that maturation of inhibition prevent the onset of critical periods, while those that enhance inhibition early in development cause a precocious critical period. Once inhibition has matured, reducing inhibition acutely can restore the capacity for ocular dominance plasticity. Manipulations that delay or accelerate the onset of the critical period for visual cortical plasticity, dark rearing and benzodiazepine application, also disrupt receptive field properties (see previous section), suggesting that inhibitory neurons play a fundamental role in the development of sensory cortex under normal conditions.

## Sensory alteration induced plasticity at inhibitory synapses

Alterations in sensory experience are not only dependent upon cortical inhibitory tone to induce plasticity; they also induce specific plastic changes at inhibitory synapses. Altering sensory drive engages inhibitory plasticity differently depending on the age of the animal, the nature of the change in sensory drive, and the specific synapses examined. For example, monocular lid suture induces rapid changes in inhibitory synaptic strength and drive, whether the lid is sutured before or after eye opening (Maffei et al., 2004, 2006, 2010; Wang et al., 2011; Kuhlman et al., 2013; Wang and Maffei, 2014). However, brief monocular lid suture before eye opening decreases drive from FS neurons onto pyramidal neurons in layer 4 of V1 (Maffei et al., 2004), whereas lid suture of the same duration started during the critical period potentiates FS to pyramidal neuron synapses in layer 4, both pre- and post-synaptically (Maffei et al., 2006; Nahmani and Turrigiano, 2014).

Removing a single row of whiskers during the first 2–3 postnatal weeks leads to an early potentiation of whisker responses in layer 2/3 of S1 after 3 days of deprivation followed by a loss of responses after 5–10 days (Drew and Feldman, 2009; Li et al., 2014a). Responses in layer 4 are not changed by this manipulation (Drew and Feldman, 2009; Li et al., 2014a). Different from lid suture, removing a single row of whiskers induces potentiation of FS to pyramidal neuron synapses in layer 2/3, where sensory plasticity is expressed (House et al., 2011).

In adult animals one of the main effects of sensory manipulation is the induction of structural changes in inhibitory neurons. A reduced number of dendritic spines has been reported in a subpopulation of inhibitory neurons following adult monocular retinal lesioning (Keck et al., 2011), suggesting that they may become less effectively activated by sensory stimuli. In addition, monocular lid suture in adult animals leads to a decrease in the number of inhibitory inputs onto layer 2/3 pyramidal neurons (van Versendaal et al., 2012), and the fraction of dynamic inhibitory synapses onto layer 2/3 pyramidal neuron spines and shafts increases (Chen et al., 2012). Thus, in adult V1 unilateral manipulation of visual drive induces a rapid restructuring of inhibitory inputs, possibly leading to an overall decreased inhibitory drive onto layer 2/3 pyramidal neurons.

While pharmacologically manipulating inhibition can change the potential for experience-dependent plasticity, global changes in circuit excitability may not be the ideal mechanism for fine-scale refinement of cortical circuit connectivity. Fine scale circuit refinement may require local, possibly synapse specific, regulation of inhibition. The age, layer and cell type specific changes in inhibitory synapses observed following monocular lid suture could provide highly specific modulation of inhibition. The layer 4 FS to pyramidal neuron synapses that are potentiated rapidly following monocular lid suture are sensitive to benzodiazepines, suggesting that they could play a role in critical period regulation (Maffei et al., 2006; Katagiri et al., 2007; Wang and Maffei, 2014). Induction of LTPi at these synapses, either through lid suture or acutely, switches the sign of plasticity induced at pyramidal neuron recurrent synapses by slow wave stimulation from long term potentiation of excitation (LTPe) to long term depression of excitation (LTDe) (Wang et al., 2012; Wang and Maffei, 2014). Acute depotentiation of the FS to pyramidal neuron synapses following lid suture restores the capacity for induction of LTPe, demonstrating that the control of inhibitory plasticity over the capacity for plasticity at excitatory synapses is modulated by experience (Wang and Maffei, 2014). While synapse specific, experience-dependent inhibitory control over excitatory plasticity has only been shown to exist in one microcircuit, this finding raises the intriguing possibility that plasticity at inhibitory synapses may control the expression of other forms of plasticity throughout cortex.

Experience-dependent reductions in inhibitory drive have been proposed to have a homeostatic role in contributing to the maintenance of neuronal activity in the face of altered sensory drive (Maffei et al., 2004; Maffei and Turrigiano, 2008b; Yang et al., 2011; Shao et al., 2013). Lid suture prior to the critical period decreases inhibitory synaptic strength onto excitatory neurons in layer 4 (Maffei et al., 2004). This homeostatic plasticity could be detected as a decrease in the spontaneous inhibitory drive and an increase in the spontaneous excitatory drive onto pyramidal neurons (Maffei et al., 2004). The induction of homeostatic inhibitory plasticity by monocular lid suture is developmentally regulated and shows layer specificity (Maffei et al., 2004; Maffei and Turrigiano, 2008b).

High decibel, high frequency sounds can lead to selective high frequency hearing loss, reorganization of the frequency map in A1 and tinnitus (Yang et al., 2011). Selective hearing loss leads to decreases in spontaneous inhibitory events and tonic inhibition thought to compensate for decreased sensory drive (Yang et al., 2011). Pharmacologically enhancing inhibition successfully eliminates tinnitus, while directly reducing excitation does not (Yang et al., 2011).

Whisker removal during a critical period for S1 plasticity reduces feedforward excitatory drive from layer 4 onto both layer 2/3 FS and pyramidal neurons, likely decreasing the activation of layer 2/3 by sensory input (House et al., 2011; Li et al., 2014a). This reduction in feedforward drive is compensated for by a robust decrease in the ability of layer 2/3 pyramidal neurons to recruit recurrent inhibition (Shao et al., 2013). These results coupled with the finding of FS to pyramidal neuron synaptic potentiation in layer 2/3 (House et al., 2011) suggest that a combination of homeostatic and Hebbian plasticity mechanisms contribute to rewiring the superficial layers of the barrel cortex.

Different forms of inhibitory synaptic plasticity can be engaged to refine or control local circuits. Despite widespread connectivity (Fino and Yuste, 2011; Packer and Yuste, 2011), the diversity of inhibitory neurons, and the selective sensitivity of distinct inhibitory synapses make them well suited to contribute to the maintenance of network excitability, large scale regulation of sensory cortical circuits and fine scale experience-dependent refinement of local microcircuits.

## Inhibition and changes in responses following sensory alterations

Understanding how changes in GABAergic neurons and their synapses contribute to changing sensory responses and maintaining homeostasis of firing rates in the face of altered sensory drive will be essential to pushing the field of experience-dependent plasticity forward. Recent work has shown that a brief unilateral reduction in visual drive strongly modulates the binocularity of FS inhibitory neurons. While FS inhibitory neurons are normally more binocular than pyramidal neurons (Swadlow, 1988; Yazaki-Sugiyama et al., 2009; Aton et al., 2013), soon after the onset of monocular lid suture or eye patching, layer 2/3 FS neurons are more strongly driven by the closed eye (Yazaki-Sugiyama et al., 2009; Aton et al., 2013; but see Kuhlman et al., 2013), although their spontaneous firing frequency is reduced (Yazaki-Sugiyama et al., 2009; Aton et al., 2013; Hengen et al., 2013). This increase in FS responsiveness to the deprived eye could contribute to reducing excitatory neuron responsiveness to that eye (Yazaki-Sugiyama et al., 2009). Interestingly, if the unilateral deprivation is maintained for several days both changes in ocular dominance of layer 2/3 FS neurons (Yazaki-Sugiyama et al., 2009; Aton et al., 2013; Kuhlman et al., 2013) and their spontaneous firing frequency are reversed (Aton et al., 2013; Hengen et al., 2013; but see Yazaki-Sugiyama et al., 2009). In layer 4 pyramidal neurons of mice, brief monocular lid suture similarly decreases both peak evoked excitatory and inhibitory conductances, and longer deprivation selectively reduces inhibition driven by the open eye (Ma et al., 2013). Differently, in rats longer monocular lid suture reduces excitation and inhibition driven by the deprived eye equally (Iurilli et al., 2013). Together, these results suggest that other mechanisms, such as changes in thalamocortical drive (Shatz and Stryker, 1978; LeVay et al., 1980; Khibnik et al., 2010; Wang et al., 2013b) may cooperate with altered intracortical inhibition to mediate the functional effects of sensory deprivation.

At first glance, studies showing that monocular lid suture does not affect the ratio of excitatory and inhibitory inputs to layer 4 pyramidal neurons (Iurilli et al., 2013; Ma et al., 2013) appear contradictory to the hypothesis that potentiation of inhibition directly suppresses visual responses (Maffei et al., 2006). However, many questions still exist. Visual stimuli used in these experiments were either moving bars or drifting gratings presented over seconds, or noise stimuli presented for several hundred milliseconds. Stimuli were presented only to one eye at a time. Additionally, only peak responses obtained during stimuli presented at peak orientation were examined (Iurilli et al., 2013; Ma et al., 2013). However, FS neurons in visual cortex have response properties that make them ideally suited to modulate extremely rapid visual responses to stimuli presented binocularly (Swadlow and Weyand, 1987; Swadlow, 1988; Yazaki-Sugiyama et al., 2009; Kameyama et al., 2010; Cardin, 2012; Aton et al., 2013; Zhuang et al., 2013). Additionally, their broad tuning raises the possibility that they could contribute to changes in orientation tuning or response suppression following lid suture. Alternatively, changes in patterns of FS neuron activity and synaptic strength could gate activity at other neurons' synapses (Levelt and Hübener, 2012; Wang and Maffei, 2014). Finally, recordings of somatic inhibitory conductances are unable to resolve differential contributions from interneuron subgroup: changes in input from one group may be offset by opposite changes in input from other groups. A recent study examining changes in excitatory and inhibitory conductances following whisker removal underscores the difficulty in interpreting results using this technique (Li et al., 2014a). Short and long durations of whisker trimming reduce evoked excitatory and inhibitory drive, but both decrease inhibition more than excitation. Interestingly, brief whisker trimming increases evoked spiking responses, while longer removal decreases responses (Li et al., 2014a). Thus, additional circuit mechanisms beyond evoked somatic excitatory and inhibitory balance must contribute to this change in responsiveness.

Two particularly exciting studies have shed light on possible roles of inhibitory plasticity in modulating sensory responses. In addition to reducing inhibition in layer 2/3, whisker trimming significantly reduces gamma oscillations in the circuit driven by the deprived whisker (Shao et al., 2013), suggesting that sensory changes in inhibitory drive may modulate network state. Whether reducing evoked gamma oscillations *in vivo* favors the induction of plasticity is not known. Like lid suture, long term monocular strabismus disrupts ocular dominance (Hubel and Wiesel, 1965), and strabismus leads to binocular suppression, whereby monocular stimuli elicit stronger responses than binocular stimuli (Sengpiel and Blakemore, 1994; Sengpiel et al., 1994). Strabismus was shown to induce binocular suppression through a selective increase in inhibitory drive during binocular stimulation (Scholl et al., 2013). These results suggest that changes in cortical inhibition induced by sensory experience may alter sensory processing in more subtle and complex ways than simply repressing unilateral deprived organ (whisker or eye) responses to optimal stimuli.

Changes in inhibitory drive are certainly not the sole mechanism at play in the experience-dependent rewiring of sensory cortex, as trophic factors, excitatory synapses, neuron intrinsic properties and extracellular matrix interactions have all been shown to contribute to the altered visual response properties and ocular dominance (Berardi et al., 2003; Levelt and Hübener, 2012). Determining how inhibitory neurons and their plasticity contribute to cortical function will require a more comprehensive understanding of how specific subclasses of inhibitory neurons contribute to sensory responses. Techniques that isolate the specific contribution of interneuron groups will need to be combined with detailed analyses of circuit and cellular properties. Many different mechanisms of GABAergic plasticity have been identified (see Castillo et al., 2011; Maffei, 2011). To manipulate these specific forms of inhibitory plasticity, it will be necessary to first identify the parameter space for their induction and expression. Specific mechanisms can then be manipulated to assess their roles in modulating neurons' response properties.

## Conclusions

The maturation and plasticity of GABAergic neurons play fundamental roles in the development of sensory cortex. Inhibitory synaptic transmission and plasticity may affect critical period timing, specific receptive field properties, spike timing and other temporal aspects of coding, gain, neural synchrony, spontaneous and evoked firing rates, the capacity for plasticity at other synapses, and contribute to the homeostasis of circuit excitability. The role of all the distinct population of inhibitory neurons in the experience-dependent reorganization of cortical circuits has not been fully explored, but there is sufficient evidence to show that different inhibitory neuron types may be engaged to modulate different aspects of neuron response properties. The challenge ahead lies in identifying the distinct roles of inhibitory neurons in cortical circuits and in determining how their plasticity contributes to modulating other neurons' response properties, connectivity, and capacity for plasticity. Development of new tools and experimental approaches to selectively manipulate specific mechanisms of inhibitory plasticity will be instrumental for determining their role in sensory cortex development and function.

### Conflict of interest statement

The authors declare that the research was conducted in the absence of any commercial or financial relationships that could be construed as a potential conflict of interest.

## References

[B1] Adesnik H. Bruns W. Taniguchi H. Huang Z. J. Scanziani M. (2012). A neural circuit for spatial summation in visual cortex. Nature 490, 226–231 10.1038/nature11526 23060193 PMC3621107

[B2] Adesnik H. Scanziani M. (2010). Lateral competition for cortical space by layer-specific horizontal circuits. Nature 464, 1155–1160 10.1038/nature08935 20414303 PMC2908490

[B3] Akgul G. Wollmuth L. P. (2013). Synapse-associated protein 97 regulates the membrane properties of fast-spiking parvalbumin interneurons in the visual cortex. J. Neurosci. 33, 12739–12750 10.1523/JNEUROSCI.0040-13.2013 23904610 PMC3728686

[B4] Ali A. B. Thomson A. M. (2008). Synaptic alpha 5 subunit-containing GABAA receptors mediate IPSPs elicited by dendrite-preferring cells in rat neocortex. Cereb. Cortex 18, 1260–1271 10.1093/cercor/bhm160 17951598

[B5] Amitai Y. (2001). Thalamocortical synaptic connections: efficacy, modulation, inhibition and plasticity. Rev. Neurosci. 12, 159–173 10.1515/REVNEURO.2001.12.2.159 11392456

[B6] Anderson J. S. Carandini M. Ferster D. (2000). Orientation tuning of input conductance, excitation, and inhibition in cat primary visual cortex. J. Neurophysiol. 84, 909–926 10938316 10.1152/jn.2000.84.2.909

[B7] Armstrong-James M. Welker E. Callahan C. A. (1993). The contribution of NMDA and non-NMDA receptors to fast and slow transmission of sensory information in the rat SI barrel cortex. J. Neurosci. 13, 2149–2160 8097531 10.1523/JNEUROSCI.13-05-02149.1993 PMC6576573

[B8] Ascoli G. A. Alonso-Nanclares L. Anderson S. A. Barrionuevo G. Benavides-Piccione R. Burkhalter A. (2008). Petilla terminology: nomenclature of features of GABAergic interneurons of the cerebral cortex. Nat. Rev. Neurosci. 9, 557–568 10.1038/nrn2402 18568015 PMC2868386

[B10] Atallah B. V. Bruns W. Carandini M. Scanziani M. (2012). Parvalbumin-expressing interneurons linearly transform cortical responses to visual stimuli. Neuron 73, 159–170 10.1016/j.neuron.2011.12.013 22243754 PMC3743079

[B9] Atencio C. A. Schreiner C. E. (2008). Spectrotemporal processing differences between auditory cortical fast-spiking and regular-spiking neurons. J. Neurosci. 28, 3897–3910 10.1523/JNEUROSCI.5366-07.2008 18400888 PMC2474630

[B11] Aton S. J. Broussard C. Dumoulin M. Seibt J. Watson A. Coleman T. (2013). Visual experience and subsequent sleep induce sequential plastic changes in putative inhibitory and excitatory cortical neurons. Proc. Natl. Acad. Sci. U.S.A. 110, 3101–3106 10.1073/pnas.1208093110 23300282 PMC3581875

[B320] Azouz R. Gray C. M. Nowak L. G. McCormick D. A. (1997). Physiological properties of inhibitory interneurons in cat striate cortex. Cereb. Cortex 7, 534–545 10.1093/cercor/7.6.534 9276178

[B12] Barkat T. R. Polley D. B. Hensch T. K. (2011). A critical period for auditory thalamocortical connectivity. Nat. Neurosci. 14, 1189–1194 10.1038/nn.2882 21804538 PMC3419581

[B13] Baroncelli L. Sale A. Viegi A. Maya Vetencourt J. F. De Pasquale R. Baldini S. (2010). Experience-dependent reactivation of ocular dominance plasticity in the adult visual cortex. Exp. Neurol. 226, 100–109 10.1016/j.expneurol.2010.08.009 20713044

[B14] Beierlein M. Gibson J. R. Connors B. W. (2003). Two dynamically distinct inhibitory networks in layer 4 of the neocortex. J. Neurophysiol. 90, 2987–3000 10.1152/jn.00283.2003 12815025

[B15] Ben-Ari Y. Khalilov I. Kahle K. T. Cherubini E. (2012). The GABA excitatory/inhibitory shift in brain maturation and neurological disorders. Neuroscientist 18, 467–486 10.1177/1073858412438697 22547529

[B16] Ben-Ari Y. Khalilov I. Represa A. Gozlan H. (2004). Interneurons set the tune of developing networks. Trends Neurosci. 27, 422–427 10.1016/j.tins.2004.05.002 15219742

[B17] Benevento L. A. Bakkum B. W. Port J. D. Cohen R. S. (1992). The effects of dark-rearing on the electrophysiology of the rat visual cortex. Brain Res. 572, 198–207 10.1016/0006-8993(92)90470-T 1611513

[B18] Benke D. Möhler H. (2006). Benzodiazepine binding to GABA(A) receptors. Curr. Protoc. Pharmacol. Chapter 1: Unit 1.16. 10.1002/0471141755.ph0116s35 22294164

[B19] Berardi N. Pizzorusso T. Ratto G. M. Maffei L. (2003). Molecular basis of plasticity in the visual cortex. Trends Neurosci. 26, 369–378 10.1016/S0166-2236(03)00168-1 12850433

[B20] Bloodgood B. L. Sharma N. Browne H. A. Trepman A. Z. Greenberg M. E. (2013). The activity-dependent transcription factor NPAS4 regulates domain-specific inhibition. Nature 503, 121–125 10.1038/nature12743 24201284 PMC4169177

[B21] Blue M. E. Parnavelas J. G. (1983). The formation and maturation of synapses in the visual cortex of the rat. II. Quantitative analysis. J. Neurocytol. 12, 697–712 10.1007/BF01181531 6619907

[B22] Bonin V. Histed M. H. Yurgenson S. Reid R. C. (2011). Local diversity and fine-scale organization of receptive fields in mouse visual cortex. J. Neurosci. 31, 18506–18521 10.1523/JNEUROSCI.2974-11.2011 22171051 PMC3758577

[B23] Borg-Graham L. J. Monier C. Frégnac Y. (1998). Visual input evokes transient and strong shunting inhibition in visual cortical neurons. Nature 393, 369–373 10.1038/30735 9620800

[B24] Bosman L. W. Rosahl T. W. Brussaard A. B. (2002). Neonatal development of the rat visual cortex: synaptic function of GABAA receptor alpha subunits. J. Physiol. 545, 169–181 10.1113/jphysiol.2002.026534 12433958 PMC2290648

[B25] Bruno R. M. Simons D. J. (2002). Feedforward mechanisms of excitatory and inhibitory cortical receptive fields. J. Neurosci. 22, 10966–10975 12486192 10.1523/JNEUROSCI.22-24-10966.2002 PMC6758449

[B26] Cardin J. A. (2012). Dissecting local circuits *in vivo*: integrated optogenetic and electrophysiology approaches for exploring inhibitory regulation of cortical activity. J. Physiol. Paris 106, 104–111 10.1016/j.jphysparis.2011.09.005 21958624 PMC3277809

[B27] Cardin J. A. Carlén M. Meletis K. Knoblich U. Zhang F. Deisseroth K. (2009). Driving fast-spiking cells induces gamma rhythm and controls sensory responses. Nature 459, 663–667 10.1038/nature08002 19396156 PMC3655711

[B28] Cardin J. A. Palmer L. A. Contreras D. (2005). Stimulus-dependent gamma (30-50 Hz) oscillations in simple and complex fast rhythmic bursting cells in primary visual cortex. J. Neurosci. 25, 5339–5350 10.1523/JNEUROSCI.0374-05.2005 15930382 PMC3034157

[B29] Cardin J. A. Palmer L. A. Contreras D. (2007). Stimulus feature selectivity in excitatory and inhibitory neurons in primary visual cortex. J. Neurosci. 27, 10333–10344 10.1523/JNEUROSCI.1692-07.2007 17898205 PMC3025280

[B30] Carmignoto G. Vicini S. (1992). Activity-dependent decrease in NMDA receptor responses during development of the visual cortex. Science 258, 1007–1011 10.1126/science.1279803 1279803

[B31] Caspary D. M. Ling L. Turner J. G. Hughes L. F. (2008). Inhibitory neurotransmission, plasticity and aging in the mammalian central auditory system. J. Exp. Biol. 211, 1781–1791 10.1242/jeb.013581 18490394 PMC2409121

[B32] Castillo P. E. Chiu C. Q. Carroll R. C. (2011). Long-term plasticity at inhibitory synapses. Curr. Opin. Neurobiol. 21, 328–338 10.1016/j.conb.2011.01.006 21334194 PMC3092861

[B33] Cauli B. Audinat E. Lambolez B. Angulo M. C. Ropert N. Tsuzuki K. (1997). Molecular and physiological diversity of cortical nonpyramidal cells. J. Neurosci. 17, 3894–3906 9133407 10.1523/JNEUROSCI.17-10-03894.1997 PMC6573690

[B34] Cauli B. Porter J. T. Tsuzuki K. Lambolez B. Rossier J. Quenet B. (2000). Classification of fusiform neocortical interneurons based on unsupervised clustering. Proc. Natl. Acad. Sci. U.S.A. 97, 6144–6149 10.1073/pnas.97.11.6144 10823957 PMC18572

[B35] Chandra D. Jia F. Liang J. Peng Z. Suryanarayanan A. Werner D. F. (2006). GABAA receptor alpha 4 subunits mediate extrasynaptic inhibition in thalamus and dentate gyrus and the action of gaboxadol. Proc. Natl. Acad. Sci. U.S.A. 103, 15230–15235 10.1073/pnas.0604304103 17005728 PMC1578762

[B36] Chattopadhyaya B. Di Cristo G. Higashiyama H. Knott G. W. Kuhlman S. J. Welker E. (2004). Experience and activity-dependent maturation of perisomatic GABAergic innervation in primary visual cortex during a postnatal critical period. J. Neurosci. 24, 9598–9611 10.1523/JNEUROSCI.1851-04.2004 15509747 PMC6730138

[B37] Chen J. L. Villa K. L. Cha J. W. So P. T. Kubota Y. Nedivi E. (2012). Clustered dynamics of inhibitory synapses and dendritic spines in the adult neocortex. Neuron 74, 361–373 10.1016/j.neuron.2012.02.030 22542188 PMC3340582

[B38] Chen X. J. Rasch M. J. Chen G. Ye C. Q. Wu S. Zhang X. H. (2014). Binocular input coincidence mediates critical period plasticity in the mouse primary visual cortex. J. Neurosci. 34, 2940–2955 10.1523/JNEUROSCI.2640-13.2014 24553935 PMC6608519

[B39] Cherubini E. Gaiarsa J. L. Ben-Ari Y. (1991). GABA: an excitatory transmitter in early postnatal life. Trends Neurosci. 14, 515–519 10.1016/0166-2236(91)90003-D 1726341

[B40] Chittajallu R. Isaac J. T. (2010). Emergence of cortical inhibition by coordinated sensory-driven plasticity at distinct synaptic loci. Nat. Neurosci. 13, 1240–1248 10.1038/nn.2639 20871602 PMC2950257

[B41] Chittajallu R. Pelkey K. A. Mcbain C. J. (2013). Neurogliaform cells dynamically regulate somatosensory integration via synapse-specific modulation. Nat. Neurosci. 16, 13–15 10.1038/nn.3284 23222912 PMC4132638

[B42] Ciceri G. Dehorter N. Sols I. Huang Z. J. Maravall M. Marín O. (2013). Lineage-specific laminar organization of cortical GABAergic interneurons. Nat. Neurosci. 16, 1199–1210 10.1038/nn.3485 23933753

[B43] Corlew R. Wang Y. Ghermazien H. Erisir A. Philpot B. D. (2007). Developmental switch in the contribution of presynaptic and postsynaptic NMDA receptors to long-term depression. J. Neurosci. 27, 9835–9845 10.1523/JNEUROSCI.5494-06.2007 17855598 PMC2905826

[B44] Cottam J. C. Smith S. L. Häusser M. (2013). Target-specific effects of somatostatin-expressing interneurons on neocortical visual processing. J. Neurosci. 33, 19567–19578 10.1523/JNEUROSCI.2624-13.2013 24336721 PMC3858626

[B45] Crochet S. Poulet J. F. Kremer Y. Petersen C. C. (2011). Synaptic mechanisms underlying sparse coding of active touch. Neuron 69, 1160–1175 10.1016/j.neuron.2011.02.022 21435560

[B46] Cruikshank S. J. Lewis T. J. Connors B. W. (2007). Synaptic basis for intense thalamocortical activation of feedforward inhibitory cells in neocortex. Nat. Neurosci. 10, 462–468 17334362 10.1038/nn1861

[B47] Cynader M. Mitchell D. E. (1980). Prolonged sensitivity to monocular deprivation in dark-reared cats. J. Neurophysiol. 43, 1026–1040 7359175 10.1152/jn.1980.43.4.1026

[B48] DeFelipe J. López-Cruz P. L. Benavides-Piccione R. Bielza C. Larrañaga P. Anderson S. (2013). New insights into the classification and nomenclature of cortical GABAergic interneurons. Nat. Rev. Neurosci. 14, 202–216 10.1038/nrn3444 23385869 PMC3619199

[B49] de la Rocha J. Marchetti C. Schiff M. Reyes A. D. (2008). Linking the response properties of cells in auditory cortex with network architecture: cotuning versus lateral inhibition. J. Neurosci. 28, 9151–9163 10.1523/JNEUROSCI.1789-08.2008 18784296 PMC2729467

[B50] Di Cristo G. Chattopadhyaya B. Kuhlman S. J. Fu Y. Belanger M. C. Wu C. Z. (2007). Activity-dependent PSA expression regulates inhibitory maturation and onset of critical period plasticity. Nat. Neurosci. 10, 1569–1577 10.1038/nn2008 18026099

[B51] Dorrn A. L. Yuan K. Barker A. J. Schreiner C. E. Froemke R. C. (2010). Developmental sensory experience balances cortical excitation and inhibition. Nature 465, 932–936 10.1038/nature09119 20559387 PMC2888507

[B52] Drew P. J. Feldman D. E. (2009). Intrinsic signal imaging of deprivation-induced contraction of whisker representations in rat somatosensory cortex. Cereb. Cortex 19, 331–348 10.1093/cercor/bhn085 18515797 PMC2638790

[B53] Espinosa J. S. Stryker M. P. (2012). Development and plasticity of the primary visual cortex. Neuron 75, 230–249 10.1016/j.neuron.2012.06.009 22841309 PMC3612584

[B54] Eyre M. D. Renzi M. Farrant M. Nusser Z. (2012). Setting the time course of inhibitory synaptic currents by mixing multiple GABA(A) receptor alpha subunit isoforms. J. Neurosci. 32, 5853–5867 10.1523/JNEUROSCI.6495-11.2012 22539847 PMC3348502

[B55] Fagiolini M. Fritschy J. M. Löw K. Möhler H. Rudolph U. Hensch T. K. (2004). Specific GABAA circuits for visual cortical plasticity. Science 303, 1681–1683 10.1126/science.1091032 15017002

[B56] Fagiolini M. Hensch T. K. (2000). Inhibitory threshold for critical-period activation in primary visual cortex. Nature 404, 183–186 10.1038/35004582 10724170

[B57] Fagiolini M. Pizzorusso T. Berardi N. Domenici L. Maffei L. (1994). Functional postnatal development of the rat primary visual cortex and the role of visual experience: dark rearing and monocular deprivation. Vision Res. 34, 709–720 10.1016/0042-6989(94)90210-0 8160387

[B58] Felmy F. Schneggenburger R. (2004). Developmental expression of the Ca2+-binding proteins calretinin and parvalbumin at the calyx of held of rats and mice. Eur. J. Neurosci. 20, 1473–1482 10.1111/j.1460-9568.2004.03604.x 15355314

[B59] Ferster D. (1988). Spatially opponent excitation and inhibition in simple cells of the cat visual cortex. J. Neurosci. 8, 1172–1180 3357015 10.1523/JNEUROSCI.08-04-01172.1988 PMC6569253

[B60] Finnerty G. T. Roberts L. S. Connors B. W. (1999). Sensory experience modifies the short-term dynamics of neocortical synapses. Nature 400, 367–371 10.1038/22553 10432115

[B61] Fino E. Packer A. M. Yuste R. (2013). The logic of inhibitory connectivity in the neocortex. Neuroscientist 19, 228–237 10.1177/1073858412456743 22922685 PMC4133777

[B62] Fino E. Yuste R. (2011). Dense inhibitory connectivity in neocortex. Neuron 69, 1188–1203 10.1016/j.neuron.2011.02.025 21435562 PMC3086675

[B63] Foeller E. Celikel T. Feldman D. E. (2005). Inhibitory sharpening of receptive fields contributes to whisker map plasticity in rat somatosensory cortex. J. Neurophysiol. 94, 4387–4400 10.1152/jn.00553.2005 16162832 PMC3070316

[B64] Földy C. Aradi I. Howard A. Soltesz I. (2004). Diversity beyond variance: modulation of firing rates and network coherence by GABAergic subpopulations. Eur. J. Neurosci. 19, 119–130 10.1046/j.1460-9568.2003.03096.x 14750970

[B65] Freund T. F. Katona I. (2007). Perisomatic inhibition. Neuron 56, 33–42 10.1016/j.neuron.2007.09.012 17920013

[B66] Froemke R. C. Merzenich M. M. Schreiner C. E. (2007). A synaptic memory trace for cortical receptive field plasticity. Nature 450, 425–429 10.1038/nature06289 18004384

[B67] Gabernet L. Jadhav S. P. Feldman D. E. Carandini M. Scanziani M. (2005). Somatosensory integration controlled by dynamic thalamocortical feed-forward inhibition. Neuron 48, 315–327 10.1016/j.neuron.2005.09.022 16242411

[B68] Galarreta M. Hestrin S. (1999). A network of fast-spiking cells in the neocortex connected by electrical synapses. Nature 402, 72–75 10.1038/47029 10573418

[B69] Galarreta M. Hestrin S. (2002). Electrical and chemical synapses among parvalbumin fast-spiking GABAergic interneurons in adult mouse neocortex. Proc. Natl. Acad. Sci. U.S.A. 99, 12438–12443 10.1073/pnas.192159599 12213962 PMC129463

[B70] Gandhi S. P. Yanagawa Y. Stryker M. P. (2008). Delayed plasticity of inhibitory neurons in developing visual cortex. Proc. Natl. Acad. Sci. U.S.A. 105, 16797–16802 10.1073/pnas.0806159105 18940923 PMC2575499

[B71] Gelman D. M. Marín O. (2010). Generation of interneuron diversity in the mouse cerebral cortex. Eur. J. Neurosci. 31, 2136–2141 10.1111/j.1460-9568.2010.07267.x 20529125

[B72] Gentet L. J. Avermann M. Matyas F. Staiger J. F. Petersen C. C. (2010). Membrane potential dynamics of GABAergic neurons in the barrel cortex of behaving mice. Neuron 65, 422–435 10.1016/j.neuron.2010.01.006 20159454

[B73] Gentet L. J. Kremer Y. Taniguchi H. Huang Z. J. Staiger J. F. Petersen C. C. (2012). Unique functional properties of somatostatin-expressing GABAergic neurons in mouse barrel cortex. Nat. Neurosci. 15, 607–612 10.1038/nn.3051 22366760

[B74] Germroth P. Schwerdtfeger W. K. Buhl E. H. (1989). GABAergic neurons in the entorhinal cortex project to the hippocampus. Brain Res. 494, 187–192 10.1016/0006-8993(89)90162-5 2765919

[B75] Gianfranceschi L. Siciliano R. Walls J. Morales B. Kirkwood A. Huang Z. J. (2003). Visual cortex is rescued from the effects of dark rearing by overexpression of BDNF. Proc. Natl. Acad. Sci. U.S.A. 100, 12486–12491 10.1073/pnas.1934836100 14514885 PMC218784

[B76] Gibson J. R. Beierlein M. Connors B. W. (1999). Two networks of electrically coupled inhibitory neurons in neocortex. Nature 402, 75–79 10.1038/47035 10573419

[B77] Gidon A. Segev I. (2012). Principles governing the operation of synaptic inhibition in dendrites. Neuron 75, 330–341 10.1016/j.neuron.2012.05.015 22841317

[B78] Gielen M. C. Lumb M. J. Smart T. G. (2012). Benzodiazepines modulate GABAA receptors by regulating the preactivation step after GABA binding. J. Neurosci. 32, 5707–5715 10.1523/JNEUROSCI.5663-11.2012 22539833 PMC6703631

[B79] Gilbert C. D. (1977). Laminar differences in receptive field properties of cells in cat primary visual cortex. J. Physiol. 268, 391–421 874916 10.1113/jphysiol.1977.sp011863 PMC1283670

[B80] Gilbert C. D. Wiesel T. N. (1979). Morphology and intracortical projections of functionally characterised neurones in the cat visual cortex. Nature 280, 120–125 10.1038/280120a0 552600

[B81] Girman S. V. Sauvé Y. Lund R. D. (1999). Receptive field properties of single neurons in rat primary visual cortex. J. Neurophysiol. 82, 301–311 10400959 10.1152/jn.1999.82.1.301

[B82] Gold C. Henze D. A. Koch C. Buzsáki G. (2006). On the origin of the extracellular action potential waveform: a modeling study. J. Neurophysiol. 95, 3113–3128 10.1152/jn.00979.2005 16467426

[B83] Goldberg E. M. Jeong H. Y. Kruglikov I. Tremblay R. Lazarenko R. M. Rudy B. (2011). Rapid developmental maturation of neocortical FS cell intrinsic excitability. Cereb. Cortex 21, 666–682 10.1093/cercor/bhq138 20705896 PMC3041012

[B84] Gomes da Silva S. Doná F. da Silva Fernandes M. J. Scorza F. A. Cavalheiro E. A. Arida R. M. (2010). Physical exercise during the adolescent period of life increases hippocampal parvalbumin expression. Brain Dev. 32, 137–142 10.1016/j.braindev.2008.12.012 19168302

[B85] Gonchar Y. Turney S. Price J. L. Burkhalter A. (2002). Axo-axonic synapses formed by somatostatin-expressing GABAergic neurons in rat and monkey visual cortex. J. Comp. Neurol. 443, 1–14 10.1002/cne.1425 11793343

[B86] González-Burgos G. Krimer L. S. Povysheva N. V. Barrionuevo G. Lewis D. A. (2005). Functional properties of fast spiking interneurons and their synaptic connections with pyramidal cells in primate dorsolateral prefrontal cortex. J. Neurophysiol. 93, 942–953 10.1152/jn.00787.2004 15385591

[B87] Gordon J. A. Stryker M. P. (1996). Experience-dependent plasticity of binocular responses in the primary visual cortex of the mouse. J. Neurosci. 16, 3274–3286 8627365 10.1523/JNEUROSCI.16-10-03274.1996 PMC6579137

[B88] Graupner M. Reyes A. D. (2013). Synaptic input correlations leading to membrane potential decorrelation of spontaneous activity in cortex. J. Neurosci. 33, 15075–15085 10.1523/JNEUROSCI.0347-13.2013 24048838 PMC3776060

[B89] Gray C. M. McCormick D. A. (1996). Chattering cells: superficial pyramidal neurons contributing to the generation of synchronous oscillations in the visual cortex. Science 274, 109–113 10.1126/science.274.5284.109 8810245

[B90] Griffen T. C. Wang L. Fontanini A. Maffei A. (2013). Developmental regulation of spatio-temporal patterns of cortical circuit activation. Front. Cell. Neurosci. 6:65 10.3389/fncel.2012.00065 23316135 PMC3539829

[B91] Grinvald A. Lieke E. Frostig R. D. Gilbert C. D. Wiesel T. N. (1986). Functional architecture of cortex revealed by optical imaging of intrinsic signals. Nature 324, 361–364 10.1038/324361a0 3785405

[B92] Hagihara K. M. Ohki K. (2013). Long-term down-regulation of GABA decreases orientation selectivity without affecting direction selectivity in mouse primary visual cortex. Front. Neural Circuits 7:28 10.3389/fncir.2013.00028 23533083 PMC3607066

[B93] Haider B. Duque A. Hasenstaub A. R. Yu Y. McCormick D. A. (2007). Enhancement of visual responsiveness by spontaneous local network activity *in vivo*. J. Neurophysiol. 97, 4186–4202 10.1152/jn.01114.2006 17409168

[B94] Haider B. Hausser M. Carandini M. (2013). Inhibition dominates sensory responses in the awake cortex. Nature 493, 97–100 10.1038/nature11665 23172139 PMC3537822

[B95] Haider B. Krause M. R. Duque A. Yu Y. Touryan J. Mazer J. A. (2010). Synaptic and network mechanisms of sparse and reliable visual cortical activity during nonclassical receptive field stimulation. Neuron 65, 107–121 10.1016/j.neuron.2009.12.005 20152117 PMC3110675

[B96] Haider B. McCormick D. A. (2009). Rapid neocortical dynamics: cellular and network mechanisms. Neuron 62, 171–189 10.1016/j.neuron.2009.04.008 19409263 PMC3132648

[B97] Hanover J. L. Huang Z. J. Tonegawa S. Stryker M. P. (1999). Brain-derived neurotrophic factor overexpression induces precocious critical period in mouse visual cortex. J Neurosci 19:RC40 10559430 10.1523/JNEUROSCI.19-22-j0003.1999 PMC2424259

[B98] Harauzov A. Spolidoro M. DiCristo G. De Pasquale R. Cancedda L. Pizzorusso T. (2010). Reducing intracortical inhibition in the adult visual cortex promotes ocular dominance plasticity. J. Neurosci. 30, 361–371 10.1523/JNEUROSCI.2233-09.2010 20053917 PMC6632513

[B99] Hayama T. Noguchi J. Watanabe S. Takahashi N. Hayashi-Takagi A. Ellis-Davies G. C. (2013). GABA promotes the competitive selection of dendritic spines by controlling local Ca2+ signaling. Nat. Neurosci. 16, 1409–1416 10.1038/nn.3496 23974706 PMC4135703

[B100] Heimel J. A. van Versendaal D. Levelt C. N. (2011). The role of GABAergic inhibition in ocular dominance plasticity. Neural Plast. 2011:391763 10.1155/2011/391763 21826276 PMC3150150

[B101] Heinen K. Bosman L. W. Spijker S. van Pelt J. Smit A. B. Voorn P. (2004). GABAA receptor maturation in relation to eye opening in the rat visual cortex. Neuroscience 124, 161–171 10.1016/j.neuroscience.2003.11.004 14960348

[B102] Helm J. Akgul G. Wollmuth L. P. (2013). Subgroups of parvalbumin-expressing interneurons in layers 2/3 of the visual cortex. J. Neurophysiol. 109, 1600–1613 10.1152/jn.00782.2012 23274311 PMC3602937

[B103] Hengen K. B. Lambo M. E. Van Hooser S. D. Katz D. B. Turrigiano G. G. (2013). Firing rate homeostasis in visual cortex of freely behaving rodents. Neuron 80, 335–342 10.1016/j.neuron.2013.08.038 24139038 PMC3816084

[B104] Hensch T. K. (2004). Critical period regulation. Annu. Rev. Neurosci. 27, 549–579 10.1146/annurev.neuro.27.070203.144327 15217343

[B105] Hensch T. K. Fagiolini M. Mataga N. Stryker M. P. Baekkeskov S. Kash S. F. (1998). Local GABA circuit control of experience-dependent plasticity in developing visual cortex. Science 282, 1504–1508 10.1126/science.282.5393.1504 9822384 PMC2851625

[B106] Hensch T. K. Stryker M. P. (2004). Columnar architecture sculpted by GABA circuits in developing cat visual cortex. Science 303, 1678–1681 10.1126/science.1091031 15017001 PMC2562723

[B107] Henze D. A. Borhegyi Z. Csicsvari J. Mamiya A. Harris K. D. Buzsáki G. (2000). Intracellular features predicted by extracellular recordings in the hippocampus *in vivo*. J. Neurophysiol. 84, 390–400 10899213 10.1152/jn.2000.84.1.390

[B108] Hestrin S. Galarreta M. (2005). Electrical synapses define networks of neocortical GABAergic neurons. Trends Neurosci. 28, 304–309 10.1016/j.tins.2005.04.001 15927686

[B109] Higley M. J. Contreras D. (2006). Balanced excitation and inhibition determine spike timing during frequency adaptation. J. Neurosci. 26, 448–457 10.1523/JNEUROSCI.3506-05.2006 16407542 PMC6674406

[B110] Hirsch J. A. Alonso J. M. Reid R. C. Martinez L. M. (1998). Synaptic integration in striate cortical simple cells. J. Neurosci. 18, 9517–9528 9801388 10.1523/JNEUROSCI.18-22-09517.1998 PMC6792880

[B111] Hirsch J. A. Martinez L. M. Pillai C. Alonso J. M. Wang Q. Sommer F. T. (2003). Functionally distinct inhibitory neurons at the first stage of visual cortical processing. Nat. Neurosci. 6, 1300–1308 10.1038/nn1152 14625553

[B112] Hou Z. H. Yu X. (2013). Activity-regulated somatostatin expression reduces dendritic spine density and lowers excitatory synaptic transmission via postsynaptic somatostatin receptor 4. J. Biol. Chem. 288, 2501–2509 10.1074/jbc.M112.419051 23233668 PMC3554918

[B113] House D. R. Elstrott J. Koh E. Chung J. Feldman D. E. (2011). Parallel regulation of feedforward inhibition and excitation during whisker map plasticity. Neuron 72, 819–831 10.1016/j.neuron.2011.09.008 22153377 PMC3240806

[B114] Hu H. Cavendish J. Z. Agmon A. (2013). Not all that glitters is gold: off-target recombination in the somatostatin-IRES-Cre mouse line labels a subset of fast-spiking interneurons. Front. Neural Circuits 7:195 10.3389/fncir.2013.00195 24339803 PMC3857604

[B115] Huang S. Huganir R. L. Kirkwood A. (2013). Adrenergic gating of Hebbian spike-timing-dependent plasticity in cortical interneurons. J. Neurosci. 33, 13171–13178 10.1523/JNEUROSCI.5741-12.2013 23926270 PMC3735889

[B116] Huang Z. J. Kirkwood A. Pizzorusso T. Porciatti V. Morales B. Bear M. F. (1999). BDNF regulates the maturation of inhibition and the critical period of plasticity in mouse visual cortex. Cell 98, 739–755 10.1016/S0092-8674(00)81509-3 10499792

[B117] Hubel D. H. Wiesel T. N. (1962). Receptive fields, binocular interaction and functional architecture in the cat's visual cortex. J. Physiol. 160, 106–154 14449617 10.1113/jphysiol.1962.sp006837 PMC1359523

[B118] Hubel D. H. Wiesel T. N. (1963). Shape and arrangement of columns in cat's striate cortex. J. Physiol. 165, 559–568 13955384 10.1113/jphysiol.1963.sp007079 PMC1359325

[B119] Hubel D. H. Wiesel T. N. (1965). Binocular interaction in striate cortex of kittens reared with artificial squint. J. Neurophysiol. 28, 1041–1059 5883731 10.1152/jn.1965.28.6.1041

[B120] Hubel D. H. Wiesel T. N. (1970). The period of susceptibility to the physiological effects of unilateral eye closure in kittens. J. Physiol. 206, 419–436 5498493 10.1113/jphysiol.1970.sp009022 PMC1348655

[B121] Inan M. Crair M. C. (2007). Development of cortical maps: perspectives from the barrel cortex. Neuroscientist 13, 49–61 10.1177/1073858406296257 17229975

[B122] Isaacson J. S. Scanziani M. (2011). How inhibition shapes cortical activity. Neuron 72, 231–243 10.1016/j.neuron.2011.09.027 22017986 PMC3236361

[B123] Iurilli G. Ghezzi D. Olcese U. Lassi G. Nazzaro C. Tonini R. (2012). Sound-driven synaptic inhibition in primary visual cortex. Neuron 73, 814–828 10.1016/j.neuron.2011.12.026 22365553 PMC3315003

[B124] Iurilli G. Olcese U. Medini P. (2013). Preserved excitatory-inhibitory balance of cortical synaptic inputs following deprived eye stimulation after a saturating period of monocular deprivation in rats. PLoS ONE 8:e82044 10.1371/journal.pone.0082044 24349181 PMC3861382

[B125] Iwai Y. Fagiolini M. Obata K. Hensch T. K. (2003). Rapid critical period induction by tonic inhibition in visual cortex. J. Neurosci. 23, 6695–6702 12890762 10.1523/JNEUROSCI.23-17-06695.2003 PMC6740711

[B126] Jeong J. K. Tremere L. A. Ryave M. J. Vuong V. C. Pinaud R. (2009). Anatomical and functional organization of inhibitory circuits in the songbird auditory forebrain. J. Exp. Neurosci. 2, 43–53 20090868 PMC2808213

[B127] Kameyama K. Sohya K. Ebina T. Fukuda A. Yanagawa Y. Tsumoto T. (2010). Difference in binocularity and ocular dominance plasticity between GABAergic and excitatory cortical neurons. J. Neurosci. 30, 1551–1559 10.1523/JNEUROSCI.5025-09.2010 20107082 PMC6633807

[B128] Katagiri H. Fagiolini M. Hensch T. K. (2007). Optimization of somatic inhibition at critical period onset in mouse visual cortex. Neuron 53, 805–812 10.1016/j.neuron.2007.02.026 17359916

[B129] Katz L. C. Shatz C. J. (1996). Synaptic activity and the construction of cortical circuits. Science 274, 1133–1138 10.1126/science.274.5290.1133 8895456

[B130] Katzner S. Busse L. Carandini M. (2011). GABAA inhibition controls response gain in visual cortex. J. Neurosci. 31, 5931–5941 10.1523/JNEUROSCI.5753-10.2011 21508218 PMC3083851

[B131] Kawaguchi Y. Kubota Y. (1993). Correlation of physiological subgroupings of nonpyramidal cells with parvalbumin- and calbindinD28k-immunoreactive neurons in layer V of rat frontal cortex. J. Neurophysiol. 70, 387–396 8395585 10.1152/jn.1993.70.1.387

[B132] Kawaguchi Y. Kubota Y. (1996). Physiological and morphological identification of somatostatin- or vasoactive intestinal polypeptide-containing cells among GABAergic cell subtypes in rat frontal cortex. J. Neurosci. 16, 2701–2715 8786446 10.1523/JNEUROSCI.16-08-02701.1996 PMC6578756

[B133] Kawaguchi Y. Kubota Y. (1997). GABAergic cell subtypes and their synaptic connections in rat frontal cortex. Cereb. Cortex 7, 476–486 10.1093/cercor/7.6.476 9276173

[B134] Keck T. Scheuss V. Jacobsen R. I. Wierenga C. J. Eysel U. T. Bonhoeffer T. (2011). Loss of sensory input causes rapid structural changes of inhibitory neurons in adult mouse visual cortex. Neuron 71, 869–882 10.1016/j.neuron.2011.06.034 21903080

[B135] Kelly J. P. Van Essen D. C. (1974). Cell structure and function in the visual cortex of the cat. J. Physiol. 238, 515–547 4136579 10.1113/jphysiol.1974.sp010541 PMC1330900

[B136] Kerlin A. M. Andermann M. L. Berezovskii V. K. Reid R. C. (2010). Broadly tuned response properties of diverse inhibitory neuron subtypes in mouse visual cortex. Neuron 67, 858–871 10.1016/j.neuron.2010.08.002 20826316 PMC3327881

[B137] Khibnik L. A. Cho K. K. Bear M. F. (2010). Relative contribution of feedforward excitatory connections to expression of ocular dominance plasticity in layer 4 of visual cortex. Neuron 66, 493–500 10.1016/j.neuron.2010.04.012 20510854 PMC2917336

[B138] Kinnischtzke A. K. Sewall A. M. Berkepile J. M. Fanselow E. E. (2012). Postnatal maturation of somatostatin-expressing inhibitory cells in the somatosensory cortex of GIN mice. Front. Neural Circuits 6:33 10.3389/fncir.2012.00033 22666189 PMC3364579

[B139] Klausberger T. Roberts J. D. Somogyi P. (2002). Cell type- and input-specific differences in the number and subtypes of synaptic GABA(A) receptors in the hippocampus. J. Neurosci. 22, 2513–2521 11923416 10.1523/JNEUROSCI.22-07-02513.2002 PMC6758298

[B140] Kobayashi M. Hamada T. Kogo M. Yanagawa Y. Obata K. Kang Y. (2008). Developmental profile of GABAA-mediated synaptic transmission in pyramidal cells of the somatosensory cortex. Eur. J. Neurosci. 28, 849–861 10.1111/j.1460-9568.2008.06401.x 18691332

[B141] Kotak V. C. Fujisawa S. Lee F. A. Karthikeyan O. Aoki C. Sanes D. H. (2005). Hearing loss raises excitability in the auditory cortex. J. Neurosci. 25, 3908–3918 10.1523/JNEUROSCI.5169-04.2005 15829643 PMC1764814

[B142] Kotak V. C. Takesian A. E. Sanes D. H. (2008). Hearing loss prevents the maturation of GABAergic transmission in the auditory cortex. Cereb. Cortex 18, 2098–2108 10.1093/cercor/bhm233 18222937 PMC2517109

[B143] Kreczko A. Goel A. Song L. Lee H. K. (2009). Visual deprivation decreases somatic GAD65 puncta number on layer 2/3 pyramidal neurons in mouse visual cortex. Neural Plast. 2009:415135 10.1155/2009/415135 19503840 PMC2686200

[B144] Kuhlman S. J. Olivas N. D. Tring E. Ikrar T. Xu X. Trachtenberg J. T. (2013). A disinhibitory microcircuit initiates critical-period plasticity in the visual cortex. Nature 501, 543–546 10.1038/nature12485 23975100 PMC3962838

[B145] Kuhlman S. J. Tring E. Trachtenberg J. T. (2011). Fast-spiking interneurons have an initial orientation bias that is lost with vision. Nat. Neurosci. 14, 1121–1123 10.1038/nn.2890 21750548 PMC3164933

[B146] Kwegyir-Afful E. E. Kyriazi H. T. Simons D. J. (2013). Weaker feedforward inhibition accounts for less pronounced thalamocortical response transformation in mouse vs. rat barrels. J. Neurophysiol. 110, 2378–2392 10.1152/jn.00574.2012 23966677 PMC3841862

[B147] Kyriazi H. T. Carvell G. E. Brumberg J. C. Simons D. J. (1996). Quantitative effects of GABA and bicuculline methiodide on receptive field properties of neurons in real and simulated whisker barrels. J. Neurophysiol. 75, 547–560 8714634 10.1152/jn.1996.75.2.547

[B148] Lampl I. Reichova I. Ferster D. (1999). Synchronous membrane potential fluctuations in neurons of the cat visual cortex. Neuron 22, 361–374 10.1016/S0896-6273(00)81096-X 10069341

[B149] Lazarus M. S. Huang Z. J. (2011). Distinct maturation profiles of perisomatic and dendritic targeting GABAergic interneurons in the mouse primary visual cortex during the critical period of ocular dominance plasticity. J. Neurophysiol. 106, 775–787 10.1152/jn.00729.2010 21613595 PMC3154827

[B150] Lee S. Hjerling-Leffler J. Zagha E. Fishell G. Rudy B. (2010). The largest group of superficial neocortical GABAergic interneurons expresses ionotropic serotonin receptors. J. Neurosci. 30, 16796–16808 10.1523/JNEUROSCI.1869-10.2010 21159951 PMC3025500

[B151a] Lee S. H. Kwan A. C. Zhang S. Phoumthipphavong V. Flannery J. G. Masmanidis S. C. (2012). Activation of specific interneurons improves V1 feature selectivity and visual perception. Nature 488, 379–383 10.1038/nature11312 22878719 PMC3422431

[B151] Lee S. Kruglikov I. Huang Z. J. Fishell G. Rudy B. (2013). A disinhibitory circuit mediates motor integration in the somatosensory cortex. Nat. Neurosci. 16, 1662–1670 10.1038/nn.3544 24097044 PMC4100076

[B152] Lefort S. Gray A. C. Turrigiano G. G. (2013). Long-term inhibitory plasticity in visual cortical layer 4 switches sign at the opening of the critical period. Proc. Natl. Acad. Sci. U.S.A. 110, E4540–E4547 10.1073/pnas.1319571110 24191045 PMC3839695

[B153] Le Magueresse C. Monyer H. (2013). GABAergic interneurons shape the functional maturation of the cortex. Neuron 77, 388–405 10.1016/j.neuron.2013.01.011 23395369

[B154] Letzkus J. J. Wolff S. B. Meyer E. M. Tovote P. Courtin J. Herry C. (2011). A disinhibitory microcircuit for associative fear learning in the auditory cortex. Nature 480, 331–335 10.1038/nature10674 22158104

[B155] LeVay S. Wiesel T. N. Hubel D. H. (1980). The development of ocular dominance columns in normal and visually deprived monkeys. J. Comp. Neurol. 191, 1–51 10.1002/cne.901910102 6772696

[B156] Levelt C. N. Hübener M. (2012). Critical-period plasticity in the visual cortex. Annu. Rev. Neurosci. 35, 309–330 10.1146/annurev-neuro-061010-113813 22462544

[B157] Levy R. B. Reyes A. D. (2011). Coexistence of lateral and co-tuned inhibitory configurations in cortical networks. PLoS Comput. Biol. 7:e1002161 10.1371/journal.pcbi.1002161 21998561 PMC3188483

[B158] Li C. X. Callaway J. C. Waters R. S. (2002). Removal of GABAergic inhibition alters subthreshold input in neurons in forepaw barrel subfield (FBS) in rat first somatosensory cortex (SI) after digit stimulation. Exp. Brain Res. 145, 411–428 10.1007/s00221-002-1124-7 12172653

[B160] Li K. Xu E. (2008). The role and the mechanism of gamma-aminobutyric acid during central nervous system development. Neurosci. Bull. 24, 195–200 10.1007/s12264-008-0109-3 18500393 PMC5552538

[B161] Li L. Gainey M. A. Goldbeck J. E. Feldman D. E. (2014a). Rapid homeostasis by disinhibition during whisker map plasticity. Proc. Natl. Acad. Sci. U.S.A. 111, 1616–1621 10.1073/pnas.1312455111 24474788 PMC3910567

[B159] Li L. Y. Xiong X. R. Ibrahim L. A. Yuan W. Tao H. W. Zhang L. I. (2014b). Differential receptive field properties of parvalbumin and somatostain inhibitory neurons in mouse auditory cortex. Cereb. Cortex. [Epub ahead of print]. 10.1093/cercor/bht417 24425250 PMC4459283

[B163] Li Y. T. Ma W. P. Li L. Y. Ibrahim L. A. Wang S. Z. Tao H. W. (2012a). Broadening of inhibitory tuning underlies contrast-dependent sharpening of orientation selectivity in mouse visual cortex. J. Neurosci. 32, 16466–16477 10.1523/JNEUROSCI.3221-12.2012 23152629 PMC3548445

[B164] Li Y. T. Ma W. P. Pan C. J. Zhang L. I. Tao H. W. (2012b). Broadening of cortical inhibition mediates developmental sharpening of orientation selectivity. J. Neurosci. 32, 3981–3991 10.1523/JNEUROSCI.5514-11.2012 22442065 PMC3372461

[B165] Lin C. S. Friedlander M. J. Sherman S. M. (1979). Morphology of physiologically identified neurons in the visual cortex of the cat. Brain Res. 172, 344–348 10.1016/0006-8993(79)90544-4 466478

[B166] Liu B. H. Li P. Li Y. T. Sun Y. J. Yanagawa Y. Obata K. (2009). Visual receptive field structure of cortical inhibitory neurons revealed by two-photon imaging guided recording. J. Neurosci. 29, 10520–10532 10.1523/JNEUROSCI.1915-09.2009 19710305 PMC2779138

[B167] Liu B. H. Li P. Sun Y. J. Li Y. T. Zhang L. I. Tao H. W. (2010). Intervening inhibition underlies simple-cell receptive field structure in visual cortex. Nat. Neurosci. 13, 89–96 10.1038/nn.2443 19946318 PMC2818750

[B168] Liu B. H. Li Y. T. Ma W. P. Pan C. J. Zhang L. I. Tao H. W. (2011). Broad inhibition sharpens orientation selectivity by expanding input dynamic range in mouse simple cells. Neuron 71, 542–554 10.1016/j.neuron.2011.06.017 21835349 PMC3154747

[B169] Lorén I. Emson P. C. Fahrenkrug J. Björklund A. Alumets J. Håkanson R. (1979). Distribution of vasoactive intestinal polypeptide in the rat and mouse brain. Neuroscience 4, 1953–1976 10.1016/0306-4522(79)90068-X 394023

[B171] Ma W. P. Liu B. H. Li Y. T. Huang Z. J. Zhang L. I. Tao H. W. (2010). Visual representations by cortical somatostatin inhibitory neurons—selective but with weak and delayed responses. J. Neurosci. 30, 14371–14379 10.1523/JNEUROSCI.3248-10.2010 20980594 PMC3001391

[B170] Ma W. P. Li Y. T. Tao H. W. (2013). Downregulation of cortical inhibition mediates ocular dominance plasticity during the critical period. J. Neurosci. 33, 11276–11280 10.1523/JNEUROSCI.5598-12.2013 23825430 PMC3718365

[B172] Ma Y. Hu H. Berrebi A. S. Mathers P. H. Agmon A. (2006). Distinct subtypes of somatostatin-containing neocortical interneurons revealed in transgenic mice. J. Neurosci. 26, 5069–5082 10.1523/JNEUROSCI.0661-06.2006 16687498 PMC2020857

[B173] Maffei A. (2011). The many forms and functions of long term plasticity at GABAergic synapses. Neural Plast. 2011:254724 10.1155/2011/254724 21789285 PMC3140781

[B174] Maffei A. Lambo M. E. Turrigiano G. G. (2010). Critical period for inhibitory plasticity in rodent binocular V1. J. Neurosci. 30, 3304–3309 10.1523/JNEUROSCI.5340-09.2010 20203190 PMC2848504

[B175] Maffei A. Nataraj K. Nelson S. B. Turrigiano G. G. (2006). Potentiation of cortical inhibition by visual deprivation. Nature 443, 81–84 10.1038/nature05079 16929304

[B176] Maffei A. Nelson S. B. Turrigiano G. G. (2004). Selective reconfiguration of layer 4 visual cortical circuitry by visual deprivation. Nat. Neurosci. 7, 1353–1359 10.1038/nn1351 15543139

[B177] Maffei A. Turrigiano G. (2008a). The age of plasticity: developmental regulation of synaptic plasticity in neocortical microcircuits. Prog. Brain Res. 169, 211–223 10.1016/S0079-6123(07)00012-X 18394476

[B178] Maffei A. Turrigiano G. G. (2008b). Multiple modes of network homeostasis in visual cortical layer 2/3. J. Neurosci. 28, 4377–4384 10.1523/JNEUROSCI.5298-07.2008 18434516 PMC2655203

[B179] Markram H. Toledo-Rodriguez M. Wang Y. Gupta A. Silberberg G. Wu C. (2004). Interneurons of the neocortical inhibitory system. Nat. Rev. Neurosci. 5, 793–807 10.1038/nrn1519 15378039

[B180] Martin del Campo H. N. Measor K. R. Razak K. A. (2012). Parvalbumin immunoreactivity in the auditory cortex of a mouse model of presbycusis. Hear. Res. 294, 31–39 10.1016/j.heares.2012.08.017 23010334

[B181] Martinez L. M. Alonso J. M. Reid R. C. Hirsch J. A. (2002). Laminar processing of stimulus orientation in cat visual cortex. J. Physiol. 540, 321–333 10.1113/jphysiol.2001.012776 11927690 PMC2290204

[B182] Maya-Vetencourt J. F. Baroncelli L. Viegi A. Tiraboschi E. Castren E. Cattaneo A. (2012). IGF-1 restores visual cortex plasticity in adult life by reducing local GABA levels. Neural Plast. 2012:250421 10.1155/2012/250421 22720172 PMC3375096

[B183] McCormick D. A. (2002). Cortical and subcortical generators of normal and abnormal rhythmicity. Int. Rev. Neurobiol. 49, 99–114 10.1016/S0074-7742(02)49009-5 12040908

[B184] Melzer S. Michael M. Caputi A. Eliava M. Fuchs E. C. Whittington M. A. (2012). Long-range-projecting GABAergic neurons modulate inhibition in hippocampus and entorhinal cortex. Science 335, 1506–1510 10.1126/science.1217139 22442486

[B185] Méndez P. Bacci A. (2011). Assortment of GABAergic plasticity in the cortical interneuron melting pot. Neural Plast. 2011:976856 10.1155/2011/976856 21785736 PMC3139185

[B186] Métin C. Godement P. Imbert M. (1988). The primary visual cortex in the mouse: receptive field properties and functional organization. Exp. Brain Res. 69, 594–612 10.1007/BF00247312 3371440

[B187] Miyoshi G. Butt S. J. Takebayashi H. Fishell G. (2007). Physiologically distinct temporal cohorts of cortical interneurons arise from telencephalic Olig2-expressing precursors. J. Neurosci. 27, 7786–7798 10.1523/JNEUROSCI.1807-07.2007 17634372 PMC6672881

[B188] Miyoshi G. Hjerling-Leffler J. Karayannis T. Sousa V. H. Butt S. J. Battiste J. (2010). Genetic fate mapping reveals that the caudal ganglionic eminence produces a large and diverse population of superficial cortical interneurons. J. Neurosci. 30, 1582–1594 10.1523/JNEUROSCI.4515-09.2010 20130169 PMC2826846

[B189] Möhler H. (2006). GABA(A) receptor diversity and pharmacology. Cell Tissue Res. 326, 505–516 10.1007/s00441-006-0284-3 16937111

[B190] Monier C. Chavane F. Baudot P. Graham L. J. Frégnac Y. (2003). Orientation and direction selectivity of synaptic inputs in visual cortical neurons: a diversity of combinations produces spike tuning. Neuron 37, 663–680 10.1016/S0896-6273(03)00064-3 12597863

[B191] Moore A. K. Wehr M. (2013). Parvalbumin-expressing inhibitory interneurons in auditory cortex are well-tuned for frequency. J. Neurosci. 33, 13713–13723 10.1523/JNEUROSCI.0663-13.2013 23966693 PMC3755717

[B192] Morales B. Choi S. Y. Kirkwood A. (2002). Dark rearing alters the development of GABAergic transmission in visual cortex. J. Neurosci. 22, 8084–8090 12223562 10.1523/JNEUROSCI.22-18-08084.2002 PMC6758086

[B193] Morrison J. H. Magistretti P. J. Benoit R. Bloom F. E. (1984). The distribution and morphological characteristics of the intracortical VIP-positive cell: an immunohistochemical analysis. Brain Res. 292, 269–282 10.1016/0006-8993(84)90763-7 6362778

[B194] Mower G. D. (1991). The effect of dark rearing on the time course of the critical period in cat visual cortex. Brain Res. Dev. Brain Res. 58, 151–158 10.1016/0165-3806(91)90001-Y 2029762

[B195] Mower G. D. Christen W. G. (1985). Role of visual experience in activating critical period in cat visual cortex. J. Neurophysiol. 53, 572–589 3981230 10.1152/jn.1985.53.2.572

[B196] Murayama M. Pérez-Garci E. Nevian T. Bock T. Senn W. Larkum M. E. (2009). Dendritic encoding of sensory stimuli controlled by deep cortical interneurons. Nature 457, 1137–1141 10.1038/nature07663 19151696

[B197] Murphy E. H. Berman N. (1979). The rabbit and the cat: a comparison of some features of response properties of single cells in the primary visual cortex. J. Comp. Neurol. 188, 401–427 10.1002/cne.901880305 489801

[B198] Nahmani M. Turrigiano G. G. (2014). Deprivation-induced strengthening of presynaptic and postsynaptic inhibitory transmission in layer 4 of visual cortex during the critical period. J. Neurosci. 34, 2571–2582 10.1523/JNEUROSCI.4600-13.2014 24523547 PMC3921427

[B199] Nase G. Weishaupt J. Stern P. Singer W. Monyer H. (1999). Genetic and epigenetic regulation of NMDA receptor expression in the rat visual cortex. Eur. J. Neurosci. 11, 4320–4326 10.1046/j.1460-9568.1999.00859.x 10594657

[B200] Niell C. M. Stryker M. P. (2008). Highly selective receptive fields in mouse visual cortex. J. Neurosci. 28, 7520–7536 10.1523/JNEUROSCI.0623-08.2008 18650330 PMC3040721

[B201] Nowak L. G. Sanchez-Vives M. V. McCormick D. A. (2008). Lack of orientation and direction selectivity in a subgroup of fast-spiking inhibitory interneurons: cellular and synaptic mechanisms and comparison with other electrophysiological cell types. Cereb. Cortex 18, 1058–1078 10.1093/cercor/bhm137 17720684 PMC3136126

[B202] Nusser Z. Sieghart W. Benke D. Fritschy J. M. Somogyi P. (1996). Differential synaptic localization of two major gamma-aminobutyric acid type A receptor alpha subunits on hippocampal pyramidal cells. Proc. Natl. Acad. Sci. U.S.A. 93, 11939–11944 10.1073/pnas.93.21.11939 8876241 PMC38162

[B203] Okun M. Lampl I. (2008). Instantaneous correlation of excitation and inhibition during ongoing and sensory-evoked activities. Nat. Neurosci. 11, 535–537 10.1038/nn.2105 18376400

[B204] Oláh S. Füle M. Komlósi G. Varga C. Báldi R. Barzó P. (2009). Regulation of cortical microcircuits by unitary GABA-mediated volume transmission. Nature 461, 1278–1281 10.1038/nature08503 19865171 PMC2771344

[B205] Olcese U. Iurilli G. Medini P. (2013). Cellular and synaptic architecture of multisensory integration in the mouse neocortex. Neuron 79, 579–593 10.1016/j.neuron.2013.06.010 23850594

[B206] Oswald A. M. Doiron B. Rinzel J. Reyes A. D. (2009). Spatial profile and differential recruitment of GABAB modulate oscillatory activity in auditory cortex. J. Neurosci. 29, 10321–10334 10.1523/JNEUROSCI.1703-09.2009 19692606 PMC2761103

[B207] Oswald A. M. Reyes A. D. (2011). Development of inhibitory timescales in auditory cortex. Cereb. Cortex 21, 1351–1361 10.1093/cercor/bhq214 21068186 PMC3097987

[B208] Ozeki H. Finn I. M. Schaffer E. S. Miller K. D. Ferster D. (2009). Inhibitory stabilization of the cortical network underlies visual surround suppression. Neuron 62, 578–592 10.1016/j.neuron.2009.03.028 19477158 PMC2691725

[B209] Packer A. M. Yuste R. (2011). Dense, unspecific connectivity of neocortical parvalbumin-positive interneurons: a canonical microcircuit for inhibition? J. Neurosci. 31, 13260–13271 10.1523/JNEUROSCI.3131-11.2011 21917809 PMC3178964

[B210] Palmer L. M. Schulz J. M. Murphy S. C. Ledergerber D. Murayama M. Larkum M. E. (2012). The cellular basis of GABA(B)-mediated interhemispheric inhibition. Science 335, 989–993 10.1126/science.1217276 22363012

[B211] Parnavelas J. G. Burne R. A. Lin C. S. (1983). Distribution and morphology of functionally identified neurons in the visual cortex of the rat. Brain Res. 261, 21–29 10.1016/0006-8993(83)91279-9 6301626

[B212] Pérez-Garci E. Gassmann M. Bettler B. Larkum M. E. (2006). The GABAB1b isoform mediates long-lasting inhibition of dendritic Ca2+ spikes in layer 5 somatosensory pyramidal neurons. Neuron 50, 603–616 10.1016/j.neuron.2006.04.019 16701210

[B213] Pfeffer C. K. Xue M. He M. Huang Z. J. Scanziani M. (2013). Inhibition of inhibition in visual cortex: the logic of connections between molecularly distinct interneurons. Nat. Neurosci. 16, 1068–1076 10.1038/nn.3446 23817549 PMC3729586

[B214] Pi H. J. Hangya B. Kvitsiani D. Sanders J. I. Huang Z. J. Kepecs A. (2013). Cortical interneurons that specialize in disinhibitory control. Nature 503, 521–524 10.1038/nature12676 24097352 PMC4017628

[B215] Piëch V. Li W. Reeke G. N. Gilbert C. D. (2013). Network model of top-down influences on local gain and contextual interactions in visual cortex. Proc. Natl. Acad. Sci. U.S.A. 110, E4108–E4117 10.1073/pnas.1317019110 24101495 PMC3808648

[B216] Pinto J. G. Hornby K. R. Jones D. G. Murphy K. M. (2010). Developmental changes in GABAergic mechanisms in human visual cortex across the lifespan. Front. Cell. Neurosci. 4:16 20592950 10.3389/fncel.2010.00016 PMC2893712

[B217] Poo C. Isaacson J. S. (2009). Odor representations in olfactory cortex: “sparse” coding, global inhibition, and oscillations. Neuron 62, 850–861 10.1016/j.neuron.2009.05.022 19555653 PMC2702531

[B218] Pouille F. Watkinson O. Scanziani M. Trevelyan A. J. (2013). The contribution of synaptic location to inhibitory gain control in pyramidal cells. Physiol. Rep. 1:e00067 10.1002/phy2.67 24303159 PMC3841021

[B219] Priebe N. J. Ferster D. (2005). Direction selectivity of excitation and inhibition in simple cells of the cat primary visual cortex. Neuron 45, 133–145 10.1016/j.neuron.2004.12.024 15629708

[B220] Priebe N. J. Ferster D. (2008). Inhibition, spike threshold, and stimulus selectivity in primary visual cortex. Neuron 57, 482–497 10.1016/j.neuron.2008.02.005 18304479

[B221] Priebe N. J. Ferster D. (2012). Mechanisms of neuronal computation in mammalian visual cortex. Neuron 75, 194–208 10.1016/j.neuron.2012.06.011 22841306 PMC3477598

[B223] Razak K. A. Fuzessery Z. M. (2009). GABA shapes selectivity for the rate and direction of frequency-modulated sweeps in the auditory cortex. J. Neurophysiol. 102, 1366–1378 10.1152/jn.00334.2009 19553486 PMC2746777

[B224] Reiter H. O. Stryker M. P. (1988). Neural plasticity without postsynaptic action potentials: less-active inputs become dominant when kitten visual cortical cells are pharmacologically inhibited. Proc. Natl. Acad. Sci. U.S.A. 85, 3623–3627 10.1073/pnas.85.10.3623 3285347 PMC280266

[B225] Renart A. de la Rocha J. Bartho P. Hollender L. Parga N. Reyes A. (2010). The asynchronous state in cortical circuits. Science 327, 587–590 10.1126/science.1179850 20110507 PMC2861483

[B226] Rochefort N. L. Narushima M. Grienberger C. Marandi N. Hill D. N. Konnerth A. (2011). Development of direction selectivity in mouse cortical neurons. Neuron 71, 425–432 10.1016/j.neuron.2011.06.013 21835340

[B227] Rudy B. Fishell G. Lee S. Hjerling-Leffler J. (2011). Three groups of interneurons account for nearly 100% of neocortical GABAergic neurons. Dev. Neurobiol. 71, 45–61 10.1002/dneu.20853 21154909 PMC3556905

[B228] Runyan C. A. Schummers J. Van Wart A. Kuhlman S. J. Wilson N. R. Huang Z. J. (2010). Response features of parvalbumin-expressing interneurons suggest precise roles for subtypes of inhibition in visual cortex. Neuron 67, 847–857 10.1016/j.neuron.2010.08.006 20826315 PMC2948796

[B229] Runyan C. A. Sur M. (2013). Response selectivity is correlated to dendritic structure in parvalbumin-expressing inhibitory neurons in visual cortex. J. Neurosci. 33, 11724–11733 10.1523/JNEUROSCI.2196-12.2013 23843539 PMC3724550

[B230] Sachidhanandam S. Sreenivasan V. Kyriakatos A. Kremer Y. Petersen C. C. (2013). Membrane potential correlates of sensory perception in mouse barrel cortex. Nat. Neurosci. 16, 1671–1677 10.1038/nn.3532 24097038

[B231] Sale A. Berardi N. Maffei L. (2009). Enrich the environment to empower the brain. Trends Neurosci. 32, 233–239 10.1016/j.tins.2008.12.004 19268375

[B232] Sale A. Maya Vetencourt J. F. Medini P. Cenni M. C. Baroncelli L. De Pasquale R. (2007). Environmental enrichment in adulthood promotes amblyopia recovery through a reduction of intracortical inhibition. Nat. Neurosci. 10, 679–681 10.1038/nn1899 17468749

[B233] Samuelsen C. L. Gardner M. P. Fontanini A. (2012). Effects of cue-triggered expectation on cortical processing of taste. Neuron 74, 410–422 10.1016/j.neuron.2012.02.031 22542192 PMC3340578

[B234] Santhakumar V. Soltesz I. (2004). Plasticity of interneuronal species diversity and parameter variance in neurological diseases. Trends Neurosci. 27, 504–510 10.1016/j.tins.2004.06.002 15271499

[B235] Saraga F. Balena T. Wolansky T. Dickson C. T. Woodin M. A. (2008). Inhibitory synaptic plasticity regulates pyramidal neuron spiking in the rodent hippocampus. Neuroscience 155, 64–75 10.1016/j.neuroscience.2008.05.009 18562122

[B236] Scholl B. Gao X. Wehr M. (2010). Nonoverlapping sets of synapses drive on responses and off responses in auditory cortex. Neuron 65, 412–421 10.1016/j.neuron.2010.01.020 20159453 PMC3800047

[B237] Scholl B. Tan A. Y. Priebe N. J. (2013). Strabismus disrupts binocular synaptic integration in primary visual cortex. J. Neurosci. 33, 17108–17122 10.1523/JNEUROSCI.1831-13.2013 24155315 PMC3807032

[B238] Schuett S. Bonhoeffer T. Hübener M. (2002). Mapping retinotopic structure in mouse visual cortex with optical imaging. J. Neurosci. 22, 6549–6559 12151534 10.1523/JNEUROSCI.22-15-06549.2002 PMC6758145

[B239] Sengpiel F. Blakemore C. (1994). Interocular control of neuronal responsiveness in cat visual cortex. Nature 368, 847–850 10.1038/368847a0 8159244

[B240] Sengpiel F. Blakemore C. Kind P. C. Harrad R. (1994). Interocular suppression in the visual cortex of strabismic cats. J. Neurosci. 14, 6855–6871 7965083 10.1523/JNEUROSCI.14-11-06855.1994 PMC6577231

[B241] Shao Y. R. Isett B. R. Miyashita T. Chung J. Pourzia O. Gasperini R. J. (2013). Plasticity of recurrent l2/3 inhibition and gamma oscillations by whisker experience. Neuron 80, 210–222 10.1016/j.neuron.2013.07.026 24094112 PMC3792397

[B242] Shatz C. J. Stryker M. P. (1978). Ocular dominance in layer IV of the cat's visual cortex and the effects of monocular deprivation. J. Physiol. 281, 267–283 702379 10.1113/jphysiol.1978.sp012421 PMC1282696

[B243] Silberberg G. Markram H. (2007). Disynaptic inhibition between neocortical pyramidal cells mediated by Martinotti cells. Neuron 53, 735–746 10.1016/j.neuron.2007.02.012 17329212

[B244] Sillito A. M. (1975). The contribution of inhibitory mechanisms to the receptive field properties of neurones in the striate cortex of the cat. J. Physiol. 250, 305–329 1177144 10.1113/jphysiol.1975.sp011056 PMC1348363

[B245] Sillito A. M. (1979). Inhibitory mechanisms influencing complex cell orientation selectivity and their modification at high resting discharge levels. J. Physiol. 289, 33–53 458666 10.1113/jphysiol.1979.sp012723 PMC1281356

[B246] Sillito A. M. Kemp J. A. Patel H. (1980). Inhibitory interactions contributing to the ocular dominance of monocularly dominated cells in the normal cat striate cortex. Exp. Brain Res. 41, 1–10 10.1007/BF00236673 7461064

[B247] Simon A. Oláh S. Molnár G. Szabadics J. Tamás G. (2005). Gap-junctional coupling between neurogliaform cells and various interneuron types in the neocortex. J. Neurosci. 25, 6278–6285 10.1523/JNEUROSCI.1431-05.2005 16000617 PMC6725286

[B248] Simons D. J. (1978). Response properties of vibrissa units in rat SI somatosensory neocortex. J. Neurophysiol. 41, 798–820 660231 10.1152/jn.1978.41.3.798

[B249] Simons D. J. Carvell G. E. (1989). Thalamocortical response transformation in the rat vibrissa/barrel system. J. Neurophysiol. 61, 311–330 2918357 10.1152/jn.1989.61.2.311

[B250] Sohya K. Kameyama K. Yanagawa Y. Obata K. Tsumoto T. (2007). GABAergic neurons are less selective to stimulus orientation than excitatory neurons in layer II/III of visual cortex, as revealed by *in vivo* functional Ca2+ imaging in transgenic mice. J. Neurosci. 27, 2145–2149 10.1523/JNEUROSCI.4641-06.2007 17314309 PMC6673543

[B251] Somogyi P. (1977). A specific ‘axo-axonal’ interneuron in the visual cortex of the rat. Brain Res. 136, 345–350 10.1016/0006-8993(77)90808-3 922488

[B252] Somogyi P. Tamás G. Lujan R. Buhl E. H. (1998). Salient features of synaptic organisation in the cerebral cortex. Brain Res. Brain Res. Rev. 26, 113–135 10.1016/S0165-0173(97)00061-1 9651498

[B253] Southwell D. G. Froemke R. C. Alvarez-Buylla A. Stryker M. P. Gandhi S. P. (2010). Cortical plasticity induced by inhibitory neuron transplantation. Science 327, 1145–1148 10.1126/science.1183962 20185728 PMC3164148

[B254] Sun Y. J. Wu G. K. Liu B. H. Li P. Zhou M. Xiao Z. (2010). Fine-tuning of pre-balanced excitation and inhibition during auditory cortical development. Nature 465, 927–931 10.1038/nature09079 20559386 PMC2909826

[B255] Swadlow H. A. (1988). Efferent neurons and suspected interneurons in binocular visual cortex of the awake rabbit: receptive fields and binocular properties. J. Neurophysiol. 59, 1162–1187 3373273 10.1152/jn.1988.59.4.1162

[B256] Swadlow H. A. (1989). Efferent neurons and suspected interneurons in S-1 vibrissa cortex of the awake rabbit: receptive fields and axonal properties. J. Neurophysiol. 62, 288–308 2754479 10.1152/jn.1989.62.1.288

[B257] Swadlow H. A. Gusev A. G. (2002). Receptive-field construction in cortical inhibitory interneurons. Nat. Neurosci. 5, 403–404 10.1038/nn847 11967546

[B258] Swadlow H. A. Weyand T. G. (1987). Corticogeniculate neurons, corticotectal neurons, and suspected interneurons in visual cortex of awake rabbits: receptive-field properties, axonal properties, and effects of EEG arousal. J. Neurophysiol. 57, 977–1001 3585466 10.1152/jn.1987.57.4.977

[B259] Szentágothai J. Arbib M. A. (1974). Conceptual models of neural organization. Neurosci. Res. Program. Bull. 12, 305–510 4437759

[B260] Takesian A. E. Kotak V. C. Sanes D. H. (2010). Presynaptic GABA(B) receptors regulate experience-dependent development of inhibitory short-term plasticity. J. Neurosci. 30, 2716–2727 10.1523/JNEUROSCI.3903-09.2010 20164356 PMC3842473

[B261] Takesian A. E. Kotak V. C. Sanes D. H. (2012). Age-dependent effect of hearing loss on cortical inhibitory synapse function. J. Neurophysiol. 107, 937–947 10.1152/jn.00515.2011 22090457 PMC3289466

[B262] Takesian A. E. Kotak V. C. Sharma N. Sanes D. H. (2013). Hearing loss differentially affects thalamic drive to two cortical interneuron subtypes. J. Neurophysiol. 110, 999–1008 10.1152/jn.00182.2013 23719211 PMC3742974

[B263] Tamamaki N. Yanagawa Y. Tomioka R. Miyazaki J. Obata K. Kaneko T. (2003). Green fluorescent protein expression and colocalization with calretinin, parvalbumin, and somatostatin in the GAD67-GFP knock-in mouse. J. Comp. Neurol. 467, 60–79 10.1002/cne.10905 14574680

[B264] Tamás G. Buhl E. H. Lörincz A. Somogyi P. (2000). Proximally targeted GABAergic synapses and gap junctions synchronize cortical interneurons. Nat. Neurosci. 3, 366–371 10.1038/73936 10725926

[B265] Tamás G. Somogyi P. Buhl E. H. (1998). Differentially interconnected networks of GABAergic interneurons in the visual cortex of the cat. J. Neurosci. 18, 4255–4270 9592103 10.1523/JNEUROSCI.18-11-04255.1998 PMC6792813

[B266] Tan A. Y. Atencio C. A. Polley D. B. Merzenich M. M. Schreiner C. E. (2007). Unbalanced synaptic inhibition can create intensity-tuned auditory cortex neurons. Neuroscience 146, 449–462 10.1016/j.neuroscience.2007.01.019 17320296

[B267] Tan A. Y. Brown B. D. Scholl B. Mohanty D. Priebe N. J. (2011). Orientation selectivity of synaptic input to neurons in mouse and cat primary visual cortex. J. Neurosci. 31, 12339–12350 10.1523/JNEUROSCI.2039-11.2011 21865476 PMC3202243

[B268] Tan A. Y. Wehr M. (2009). Balanced tone-evoked synaptic excitation and inhibition in mouse auditory cortex. Neuroscience 163, 1302–1315 10.1016/j.neuroscience.2009.07.032 19628023

[B269] Tan A. Y. Zhang L. I. Merzenich M. M. Schreiner C. E. (2004). Tone-evoked excitatory and inhibitory synaptic conductances of primary auditory cortex neurons. J. Neurophysiol. 92, 630–643 10.1152/jn.01020.2003 14999047

[B270] Taniguchi H. (2014). Genetic dissection of GABAergic neural circuits in mouse neocortex. Front. Cell. Neurosci. 8:8 10.3389/fncel.2014.00008 24478631 PMC3902216

[B271] Taniguchi H. He M. Wu P. Kim S. Paik R. Sugino K. (2011). A resource of Cre driver lines for genetic targeting of GABAergic neurons in cerebral cortex. Neuron 71, 995–1013 10.1016/j.neuron.2011.07.026 21943598 PMC3779648

[B272] Taniguchi H. Lu J. Huang Z. J. (2013). The spatial and temporal origin of chandelier cells in mouse neocortex. Science 339, 70–74 10.1126/science.1227622 23180771 PMC4017638

[B273] Thomson A. M. West D. C. Wang Y. Bannister A. P. (2002). Synaptic connections and small circuits involving excitatory and inhibitory neurons in layers 2-5 of adult rat and cat neocortex: triple intracellular recordings and biocytin labelling *in vitro*. Cereb. Cortex 12, 936–953 10.1093/cercor/12.9.936 12183393

[B274] Tiao Y. C. Blakemore C. (1976). Functional organization in the visual cortex of the golden hamster. J. Comp. Neurol. 168, 459–481 10.1002/cne.901680403 939818

[B275] Tremere L. Hicks T. P. Rasmusson D. D. (2001). Expansion of receptive fields in raccoon somatosensory cortex *in vivo* by GABA(A) receptor antagonism: implications for cortical reorganization. Exp. Brain Res. 136, 447–455 10.1007/s002210000612 11291725

[B276] Van Hooser S. D. Heimel J. A. Chung S. Nelson S. B. Toth L. J. (2005). Orientation selectivity without orientation maps in visual cortex of a highly visual mammal. J. Neurosci. 25, 19–28 10.1523/JNEUROSCI.4042-04.2005 15634763 PMC6725193

[B277] van Versendaal D. Rajendran R. Saiepour M. H. Klooster J. Smit-Rigter L. Sommeijer J. P. (2012). Elimination of inhibitory synapses is a major component of adult ocular dominance plasticity. Neuron 74, 374–383 10.1016/j.neuron.2012.03.015 22542189

[B278] Vitalis T. Rossier J. (2011). New insights into cortical interneurons development and classification: contribution of developmental studies. Dev. Neurobiol. 71, 34–44 10.1002/dneu.20810 21154908

[B279] Wahle P. (1993). Differential regulation of substance P and somatostatin in Martinotti cells of the developing cat visual cortex. J. Comp. Neurol. 329, 519–538 10.1002/cne.903290408 7681071

[B280] Wang B. S. Feng L. Liu M. Liu X. Cang J. (2013a). Environmental enrichment rescues binocular matching of orientation preference in mice that have a precocious critical period. Neuron 80, 198–209 10.1016/j.neuron.2013.07.023 24012279 PMC3796011

[B281] Wang B. S. Sarnaik R. Cang J. (2010). Critical period plasticity matches binocular orientation preference in the visual cortex. Neuron 65, 246–256 10.1016/j.neuron.2010.01.002 20152130 PMC2822731

[B282] Wang L. Fontanini A. Maffei A. (2011). Visual experience modulates spatio-temporal dynamics of circuit activation. Front. Cell. Neurosci. 5:12 10.3389/fncel.2011.00012 21743804 PMC3127086

[B283] Wang L. Fontanini A. Maffei A. (2012). Experience-dependent switch in sign and mechanisms for plasticity in layer 4 of primary visual cortex. J. Neurosci. 32, 10562–10573 10.1523/JNEUROSCI.0622-12.2012 22855806 PMC3434710

[B284] Wang L. Kloc M. Gu Y. Ge S. Maffei A. (2013b). Layer-specific experience-dependent rewiring of thalamocortical circuits. J. Neurosci. 33, 4181–4191 10.1523/JNEUROSCI.4423-12.2013 23447625 PMC3613853

[B285] Wang L. Maffei A. (2014). Inhibitory plasticity dictates the sign of plasticity at excitatory synapses. J. Neurosci. 34, 1083–1093 10.1523/JNEUROSCI.4711-13.2014 24453301 PMC3898280

[B286] Wang Y. Gupta A. Toledo-Rodriguez M. Wu C. Z. Markram H. (2002). Anatomical, physiological, molecular and circuit properties of nest basket cells in the developing somatosensory cortex. Cereb. Cortex 12, 395–410 10.1093/cercor/12.4.395 11884355

[B287] Wang Y. Kakizaki T. Sakagami H. Saito K. Ebihara S. Kato M. (2009). Fluorescent labeling of both GABAergic and glycinergic neurons in vesicular GABA transporter (VGAT)-venus transgenic mouse. Neuroscience 164, 1031–1043 10.1016/j.neuroscience.2009.09.010 19766173

[B288] Wang Y. Toledo-Rodriguez M. Gupta A. Wu C. Silberberg G. Luo J. (2004). Anatomical, physiological and molecular properties of Martinotti cells in the somatosensory cortex of the juvenile rat. J. Physiol. 561, 65–90 10.1113/jphysiol.2004.073353 15331670 PMC1665344

[B289] Wehr M. Zador A. M. (2003). Balanced inhibition underlies tuning and sharpens spike timing in auditory cortex. Nature 426, 442–446 10.1038/nature02116 14647382

[B290] Wehr M. Zador A. M. (2005). Synaptic mechanisms of forward suppression in rat auditory cortex. Neuron 47, 437–445 10.1016/j.neuron.2005.06.009 16055066

[B291] Whittington M. A. Traub R. D. Kopell N. Ermentrout B. Buhl E. H. (2000). Inhibition-based rhythms: experimental and mathematical observations on network dynamics. Int. J. Psychophysiol. 38, 315–336 10.1016/S0167-8760(00)00173-2 11102670

[B292] Wiesel T. N. Hubel D. H. (1963). Single-cell responses in striate cortex of kittens deprived of vision in one eye. J. Neurophysiol. 26, 1003–1017 14084161 10.1152/jn.1963.26.6.1003

[B293] Wilent W. B. Contreras D. (2004). Synaptic responses to whisker deflections in rat barrel cortex as a function of cortical layer and stimulus intensity. J. Neurosci. 24, 3985–3998 10.1523/JNEUROSCI.5782-03.2004 15102914 PMC6729426

[B294] Wilent W. B. Contreras D. (2005). Dynamics of excitation and inhibition underlying stimulus selectivity in rat somatosensory cortex. Nat. Neurosci. 8, 1364–1370 10.1038/nn1545 16158064

[B295] Wilson N. R. Runyan C. A. Wang F. L. Sur M. (2012). Division and subtraction by distinct cortical inhibitory networks *in vivo*. Nature 488, 343–348 10.1038/nature11347 22878717 PMC3653570

[B296] Wisden W. Cope D. Klausberger T. Hauer B. Sinkkonen S. T. Tretter V. (2002). Ectopic expression of the GABA(A) receptor alpha6 subunit in hippocampal pyramidal neurons produces extrasynaptic receptors and an increased tonic inhibition. Neuropharmacology 43, 530–549 10.1016/S0028-3908(02)00151-X 12367600

[B297] Wu G. K. Arbuckle R. Liu B. H. Tao H. W. Zhang L. I. (2008). Lateral sharpening of cortical frequency tuning by approximately balanced inhibition. Neuron 58, 132–143 10.1016/j.neuron.2008.01.035 18400169 PMC2447869

[B298] Wu G. K. Li P. Tao H. W. Zhang L. I. (2006). Nonmonotonic synaptic excitation and imbalanced inhibition underlying cortical intensity tuning. Neuron 52, 705–715 10.1016/j.neuron.2006.10.009 17114053 PMC1764440

[B299] Wu G. K. Tao H. W. Zhang L. I. (2011). From elementary synaptic circuits to information processing in primary auditory cortex. Neurosci. Biobehav. Rev. 35, 2094–2104 10.1016/j.neubiorev.2011.05.004 21609731 PMC3184206

[B300] Wu X. Wu Z. Ning G. Guo Y. Ali R. Macdonald R. L. (2012). gamma-Aminobutyric acid type A (GABAA) receptor alpha subunits play a direct role in synaptic versus extrasynaptic targeting. J. Biol. Chem. 287, 27417–27430 10.1074/jbc.M112.360461 22711532 PMC3431651

[B301] Xu H. Jeong H. Y. Tremblay R. Rudy B. (2013). Neocortical somatostatin-expressing GABAergic interneurons disinhibit the thalamorecipient layer 4. Neuron 77, 155–167 10.1016/j.neuron.2012.11.004 23312523 PMC3556168

[B302] Xu H. Kotak V. C. Sanes D. H. (2010). Normal hearing is required for the emergence of long-lasting inhibitory potentiation in cortex. J. Neurosci. 30, 331–341 10.1523/JNEUROSCI.4554-09.2010 20053914 PMC2823134

[B303] Xu Q. de la Cruz E. Anderson S. A. (2003). Cortical interneuron fate determination: diverse sources for distinct subtypes? Cereb. Cortex 13, 670–676 10.1093/cercor/13.6.670 12764043

[B304] Xu X. Callaway E. M. (2009). Laminar specificity of functional input to distinct types of inhibitory cortical neurons. J. Neurosci. 29, 70–85 10.1523/JNEUROSCI.4104-08.2009 19129386 PMC2656387

[B305] Yang S. Weiner B. D. Zhang L. S. Cho S. J. Bao S. (2011). Homeostatic plasticity drives tinnitus perception in an animal model. Proc. Natl. Acad. Sci. U.S.A. 108, 14974–14979 10.1073/pnas.1107998108 21896771 PMC3169130

[B306] Yashiro K. Philpot B. D. (2008). Regulation of NMDA receptor subunit expression and its implications for LTD, LTP, and metaplasticity. Neuropharmacology 55, 1081–1094 10.1016/j.neuropharm.2008.07.046 18755202 PMC2590778

[B307] Yazaki-Sugiyama Y. Kang S. Câteau H. Fukai T. Hensch T. K. (2009). Bidirectional plasticity in fast-spiking GABA circuits by visual experience. Nature 462, 218–221 10.1038/nature08485 19907494

[B308] Yokota T. Eguchi K. Hiraba K. (2011). Functional properties of putative pyramidal neurons and inhibitory interneurons in the rat gustatory cortex. Cereb. Cortex 21, 597–606 10.1093/cercor/bhq126 20615912

[B309] Yu J. Ferster D. (2010). Membrane potential synchrony in primary visual cortex during sensory stimulation. Neuron 68, 1187–1201 10.1016/j.neuron.2010.11.027 21172618 PMC3031170

[B310] Zariwala H. A. Madisen L. Ahrens K. F. Bernard A. Lein E. S. Jones A. R. (2011). Visual tuning properties of genetically identified layer 2/3 neuronal types in the primary visual cortex of cre-transgenic mice. Front. Syst. Neurosci. 4:162 10.3389/fnsys.2010.00162 21283555 PMC3028542

[B311] Zhang L. I. Bao S. Merzenich M. M. (2001). Persistent and specific influences of early acoustic environments on primary auditory cortex. Nat. Neurosci. 4, 1123–1130 10.1038/nn745 11687817

[B312] Zhang L. I. Tan A. Y. Schreiner C. E. Merzenich M. M. (2003). Topography and synaptic shaping of direction selectivity in primary auditory cortex. Nature 424, 201–205 10.1038/nature01796 12853959

[B313] Zhang Z. Jiao Y. Y. Sun Q. Q. (2011). Developmental maturation of excitation and inhibition balance in principal neurons across four layers of somatosensory cortex. Neuroscience 174, 10–25 10.1016/j.neuroscience.2010.11.045 21115101 PMC3020261

[B314] Zheng J. J. Li S. J. Zhang X. D. Miao W. Y. Zhang D. Yao H. (2014). Oxytocin mediates early experience-dependent cross-modal plasticity in the sensory cortices. Nat. Neurosci. 17, 391–399 10.1038/nn.3634 24464043

[B315] Zhou Y. Liu B. H. Wu G. K. Kim Y. J. Xiao Z. Tao H. W. (2010). Preceding inhibition silences layer 6 neurons in auditory cortex. Neuron 65, 706–717 10.1016/j.neuron.2010.02.021 20223205 PMC2904750

[B319] Zhuang J. Stoelzel C. R. Bereshpolova Y. Huff J. M. Hei X. Alonso J. M. (2013). Layer 4 in primary visual cortex of the awake rabbit: contrasting properties of simple cells and putative feedforward inhibitory interneurons. J. Neurosci. 33, 11372–11389 10.1523/JNEUROSCI.0863-13.2013 23843510 PMC3724558

[B316] Zhu J. J. Connors B. W. (1999). Intrinsic firing patterns and whisker-evoked synaptic responses of neurons in the rat barrel cortex. J. Neurophysiol. 81, 1171–1183 10085344 10.1152/jn.1999.81.3.1171

[B317] Zhu Y. Stornetta R. L. Zhu J. J. (2004). Chandelier cells control excessive cortical excitation: characteristics of whisker-evoked synaptic responses of layer 2/3 nonpyramidal and pyramidal neurons. J. Neurosci. 24, 5101–5108 10.1523/JNEUROSCI.0544-04.2004 15175379 PMC6729194

[B318] Zhu Y. Zhu J. J. (2004). Rapid arrival and integration of ascending sensory information in layer 1 nonpyramidal neurons and tuft dendrites of layer 5 pyramidal neurons of the neocortex. J. Neurosci. 24, 1272–1279 10.1523/JNEUROSCI.4805-03.2004 14960597 PMC6730332

